# Predictive value of circulating NMR metabolic biomarkers for type 2 diabetes risk in the UK Biobank study

**DOI:** 10.1186/s12916-022-02354-9

**Published:** 2022-05-03

**Authors:** Fiona Bragg, Eirini Trichia, Diego Aguilar-Ramirez, Jelena Bešević, Sarah Lewington, Jonathan Emberson

**Affiliations:** 1 MRC Population Health Research Unit, Nuffield Department of Population Health, University of Oxford, Old Road Campus, Oxford, OX3 7LF UK; 2 Clinical Trial Service Unit & Epidemiological Studies Unit, Nuffield Department of Population Health, University of Oxford, Old Road Campus, Oxford, OX3 7LF UK; 3 UKM Medical Molecular Biology Institute (UMBI), Universiti Kebangsaan Malaysia, Kuala Lumpur, Malaysia

**Keywords:** Biomarkers, Diabetes, Metabolomics, Risk prediction

## Abstract

**Background:**

Effective targeted prevention of type 2 diabetes (T2D) depends on accurate prediction of disease risk. We assessed the role of metabolomic profiling in improving T2D risk prediction beyond conventional risk factors.

**Methods:**

Nuclear magnetic resonance (NMR) metabolomic profiling was undertaken on baseline plasma samples in 65,684 UK Biobank participants without diabetes and not taking lipid-lowering medication. Among a subset of 50,519 participants with data available on all relevant co-variates (sociodemographic characteristics, parental history of diabetes, lifestyle—including dietary—factors, anthropometric measures and fasting time), Cox regression yielded adjusted hazard ratios for the associations of 143 individual metabolic biomarkers (including lipids, lipoproteins, fatty acids, amino acids, ketone bodies and other low molecular weight metabolic biomarkers) and 11 metabolic biomarker principal components (PCs) (accounting for 90% of the total variance in individual biomarkers) with incident T2D. These 11 PCs were added to established models for T2D risk prediction among the full study population, and measures of risk discrimination (c-statistic) and reclassification (continuous net reclassification improvement [NRI], integrated discrimination index [IDI]) were assessed.

**Results:**

During median 11.9 (IQR 11.1–12.6) years’ follow-up, after accounting for multiple testing, 90 metabolic biomarkers showed independent associations with T2D risk among 50,519 participants (1211 incident T2D cases) and 76 showed associations after additional adjustment for HbA1c (false discovery rate controlled *p* < 0.01). Overall, 8 metabolic biomarker PCs were independently associated with T2D. Among the full study population of 65,684 participants, of whom 1719 developed T2D, addition of PCs to an established risk prediction model, including age, sex, parental history of diabetes, body mass index and HbA1c, improved T2D risk prediction as assessed by the c-statistic (increased from 0.802 [95% CI 0.791–0.812] to 0.830 [0.822–0.841]), continuous NRI (0.44 [0.38–0.49]) and relative (15.0% [10.5–20.4%]) and absolute (1.5 [1.0–1.9]) IDI. More modest improvements were observed when metabolic biomarker PCs were added to a more comprehensive established T2D risk prediction model additionally including waist circumference, blood pressure and plasma lipid concentrations (c-statistic, 0.829 [0.819–0.838] to 0.837 [0.831–0.848]; continuous NRI, 0.22 [0.17–0.28]; relative IDI, 6.3% [4.1–9.8%]; absolute IDI, 0.7 [0.4–1.1]).

**Conclusions:**

When added to conventional risk factors, circulating NMR-based metabolic biomarkers modestly enhanced T2D risk prediction.

**Supplementary Information:**

The online version contains supplementary material available at 10.1186/s12916-022-02354-9.

## Background

Both population-level and individual high-risk prevention approaches are essential for addressing the major and rising global public health challenge of type 2 diabetes (T2D). Fundamental to the latter is the ability to accurately predict future T2D risk. This enables targeted or *precision* prevention of the disease among individuals at high risk [1] through evidence-based lifestyle or pharmacologic interventions capable of preventing or delaying the onset of T2D [1] and ultimately its complications [2]. Existing T2D risk prediction models perform well in their ability to discriminate between individuals at low or high future risk of T2D [3–5]. However, these models are imperfect, frequently over-estimating T2D risk [6] and often lacking sufficient specificity to be of use clinically [7]. Moreover, they characteristically rely on distal risk factors and consider, at best, only limited molecular pathways. This contrasts with the classical T2D prodrome, comprising dysregulation of multiple molecular pathways over a period of many years [8].

Through metabolomic profiling, large numbers of biomarkers across multiple biological pathways—proximal and distal—can be quantified in a single measurement, capturing the consequences of genetic variation, environmental influences, and their interactions. Prospective studies have established associations of diverse circulating metabolic biomarkers (e.g. amino acids, fatty acids, hexoses, lipids) with T2D [9]. As well as providing aetiological insights, these data might feasibly contribute valuable risk prediction information. Previous studies investigating the ability of metabolomics to improve T2D risk prediction over established risk factors have, with the exception of a small number of studies [10, 11], based their findings on limited T2D cases [12–17], frequently investigating only small numbers or single subclasses of metabolic biomarkers [11–16], or have used untargeted metabolomic profiling, including unknown biomarkers [16, 17] which are less easily and expeditiously translated into clinical use. The resulting inconsistent findings leave an ongoing uncertainty regarding the value of metabolomic profiling for T2D risk prediction.

Using recently available data from the UK Biobank study, we characterise the prospective associations of circulating metabolic biomarkers, quantified using a high-throughput targeted nuclear magnetic resonance (NMR) metabolomics platform, with the risk of incident T2D, and examine whether the addition of these biomarkers to established models improves prediction of T2D risk.

## Methods

### Study population

Details of the UK Biobank (UKB) (https://www.ukbiobank.ac.uk/) study design and population have been described previously [18]. Briefly, postal invitations to participate were sent to 9.2 million adults aged 40–69 years, living in England, Wales or Scotland and registered with the UK National Health Service. A response rate of 5.5% was achieved, and 502,493 participants were enrolled.

### Data collection

The baseline survey took place between 2006 and 2010 in 22 assessment centres. Self-administered touchscreen questionnaires collected information on sociodemographic and lifestyle factors (including diet, physical activity, smoking and alcohol drinking) and personal (supplemented by verbal interview) and family medical history. Physical measurements, including blood pressure, height, weight, waist circumference (WC) and hip circumference, were undertaken using calibrated instruments with standard protocols. A non-fasting venous blood sample was collected, with the time since the last food or drink recorded. After minimal processing at assessment centres, samples were shipped to a central facility for processing and long-term storage at − 80 °C. Biochemical biomarkers were measured on stored baseline samples at a central UK Biobank laboratory between 2014 and 2017 [19]. These included HDL cholesterol and triglycerides (AU5400; Beckman Coulter) and HbA1c (VARIANT II TURBO Hemoglobin Testing System; Bio-Rad). Repeat surveys collected the same information as at baseline in addition to certain enhancements; they comprised a resurvey of ~ 20,000 participants in 2012–2013 and an ongoing survey of ~ 100,000 participants which commenced in 2014 [20, 21].

All participants consented to be followed up through linkage to health-related records. These included prior and prospective data on dates and causes of hospital admissions (Hospital Episode Statistics in England, Patient Episode Database for Wales, and Scottish Morbidity Record) and primary care clinical events and prescribing (available for ~ 45% of participants), as well as date and cause of death obtained from national death registries.

### Metabolic biomarker quantification

A high-throughput NMR metabolomics platform [22, 23] was used to undertake metabolomic profiling in baseline plasma samples from a randomly selected subset of ~ 120,000 UKB participants [24]. This simultaneously quantified 249 metabolic biomarkers (168 directly measured and 81 ratios of these), including lipids, fatty acids, amino acids, ketone bodies and other low-molecular-weight metabolic biomarkers (e.g. gluconeogenesis-related metabolites), as well as lipoprotein subclass distribution, particle size and composition. A subset of 143 (Additional file 1: Table S1) was selected for inclusion in the presented analyses, focussing on those which were directly measured and could not be inferred from other biomarkers.

### Assessment of incident type 2 diabetes status

Incident T2D status was ascertained through (i) self-report of T2D diagnosis or glucose-lowering medication use at repeat surveys; (ii) coded T2D diagnoses recorded in primary care, hospital admission or death registry data; or (iii) glucose-lowering medication prescribing in primary care data (Additional file 1: Table S2). Only those participants without diagnostic codes for other specified diabetes types (type 1/malnutrition-related/other specified diabetes) were considered to have T2D.

### Statistical analysis

The analyses excluded those with previously diagnosed diabetes of any type (based on self-report, primary care or inpatient hospital data), taking regular glucose-lowering medication (based on self-report or primary care data) or with HbA1c ≥ 6.5% (corresponding to 48 mmol/mol and consistent with undiagnosed diabetes) at the baseline survey. Those with missing or extreme NMR biomarker or covariate data (see below), or who were taking lipid-lowering medications at recruitment, were also excluded from the main analyses. This generated a ‘risk prediction population’ comprising 65,684 participants and, following the exclusion of a further 15,165 participants with missing data for additional covariates included only in association analyses, an ‘association analyses population’ of 50,519 participants (Additional file 1: Fig. S1).

All NMR biomarkers were log-transformed and standardised. Principal component analysis was then employed to reduce a large number of correlated NMR biomarkers (Additional file 1: Fig. S2) to a much smaller number of uncorrelated principal components (PCs) which retained most (> 90%) of the variance in the individual biomarkers. Cox regression among 50,519 participants in the association analyses population was used to assess the individual relevance of each NMR biomarker (and each PC) to the risk of incident T2D. First, to examine the shape of the associations, participants were grouped into baseline categories defined by quartiles of their distributions and a test of trend performed across quartiles. Subsequently, continuous analyses of each NMR biomarker (and each PC) were done to estimate the HR per 1−SD higher baseline level. Cox models were stratified by age-at-risk (5-year age groups) and sex and adjusted for assessment centre (22 centres), Townsend Deprivation Index (numeric), ethnicity (6 categories), parental history of diabetes (2 categories), smoking (4 categories), alcohol drinking (4 categories), physical activity (numeric), dietary factors (whole and refined grains, fruit, vegetables, cheese, unprocessed red meat, processed meat, non-oily and oily fish, type of spread, tea [all 4 categories], coffee [caffeinated 4 categories; decaffeinated 3 categories], dietary supplements [4 categories]), body mass index (BMI) (numeric), waist-to-hip ratio (WHR) (numeric), fasting time (numeric) and spectrometer (6 spectrometers). Participants who did not develop incident T2D were censored at the earliest of death, loss to follow-up or 31 December 2020. For significance testing, the Benjamini-Hochberg method was used to control the false discovery rate (FDR) [25]. Statistical significance was defined as FDR controlled *p* < 0.01. Sensitivity analyses examined the associations separately by age (< 55 vs ≥ 55 years) and sex and after additional adjustment for HbA1c. In addition, the impact of excluding the first 3 years of follow-up was assessed and, for the analysis of each PC, mutual adjustment for all preceding PCs.

Then, to assess whether circulating NMR biomarkers could improve the prediction of T2D risk, the selected PCs were added to the ‘traditional’ T2D risk prediction models based on Framingham risk scores for T2D [3] among 65,684 participants in the risk prediction population. Two models were assessed: a ‘concise’ model, including age (< 50, 50–64, ≥ 65 years), sex, parental history of diabetes, BMI (< 25.0, 25.0–29.9, ≥ 30.0 kg/m^2^) and HbA1c (< 6.0% vs ≥ 6.0%), and a ‘full’ model, which additionally included blood pressure (≤ 130/85 mmHg and not taking anti-hypertensive medication vs > 130/85 mmHg or taking anti-hypertensive medication), HDL cholesterol (< 1.0 vs ≥ 1.0 mmol/L in men; < 1.3 vs ≥ 1.3 mmol/L in women), triglycerides (< 1.7 vs ≥ 1.7 mmol/L), and WC (≤ 102 vs > 102 cm in men; ≤ 88 vs > 88 cm in women) [3]. The discriminatory ability of each model before and after including the PCs was assessed using Harrell’s c-statistic [26], and the likelihood ratio test was used to compare the fits of nested models (i.e. those including versus excluding the PCs). Relative and absolute integrated discrimination improvement (IDI) [27] and continuous net reclassification improvement (NRI) [28] were estimated to assess risk reclassification. To avoid model optimism, bootstrapping was used to create bias-corrected estimates and CIs for the c-statistics, IDI and NRI. To test model calibration, observed T2D event rates for absolute predicted risk deciles were plotted against their predicted event rates, and calibration slopes were estimated using a Cox regression analysis of predicted risk on observed risk. Calibration slopes and their confidence intervals were estimated from 10-fold cross-validation (pooled using inverse variance weighting). Subsequent analyses assessed the performance of the four risk prediction models solely among 13,695 participants taking lipid-lowering medications at baseline. Sensitivity analyses separately assessed their performance after replacing WC with WHR and, where appropriate, including model covariates as continuous variables.

Analyses were conducted using SAS (version 9.4) and R (version 3.6.2).

## Results

Of the original 502,493 UKB participants, a random subset of 118,036 (23%) had NMR biomarker data (Additional file 1: Fig. S1, Additional file 1: Table S3). Of these, 65,684 (56%) had no prior diabetes, were not taking lipid-lowering medication and had complete NMR biomarker (and other) data for inclusion in subsequent risk prediction analyses. The mean (SD) age of participants in this risk prediction population was 55.2 (8.0) years, and 58% (*n* = 37,849) were women (Table 1). During 0.8 million person-years of follow-up (median 11.9 [IQR 11.1–12.6]), 1719 cases of incident T2D were identified. Participants who developed T2D were more likely to be male and, at the time of recruitment, tended to be older and of lower socioeconomic status than those who did not develop T2D. They also had higher levels of adiposity, were more likely to be current regular smokers, but less likely to be current regular alcohol drinkers, and more frequently had a parental history of diabetes.

**Table 1:** Baseline characteristics of 65,684 participants in the risk prediction population by incident type 2 diabetes status

Baseline characteristics^a^	Incident type 2 diabetes		Total
	Yes	No	
**No. of participants**	1719	63,965	65,684
**Age, sex and socioeconomic factors**			
Mean age (SD), years	57.1 (7.7)	55.1 (8.0)	55.2 (8.0)
Women, %	47	58	58
Townsend Deprivation Index (SD)^b^	0.3 (1.1)	0.0 (1.0)	0.0 (1.0)
**Lifestyle factors**			
Smoking, %			
Never or occasional	54	61	61
Previous	33	32	32
Current regular	14	7	7
Alcohol drinking, %			
Never or occasional	42	25	26
Previous	5	3	3
Current regular	53	71	71
**Physical and blood-based measurements, mean (SD)**			
BMI, kg/m^2^	31.5 (5.2)	26.8 (4.5)	26.9 (4.4)
WC, cm	100 (13)	88 (12)	88 (13)
HC, cm	110 (10)	103 (9)	103 (9)
WHR	0.91 (0.08)	0.86 (0.09)	0.86 (0.09)
SBP, mmHg	142 (19)	136 (18)	136 (18)
DBP, mmHg	86 (11)	82 (10)	82 (10)
Total cholesterol, mmol/L^c^	6.0 (1.1)	5.9 (1.1)	5.9 (1.1)
LDL cholesterol, mmol/L^c^	3.8 (0.8)	3.7 (0.8)	3.7 (0.8)
HDL cholesterol, mmol/L^c^	1.3 (0.3)	1.5 (0.4)	1.5 (0.4)
Triglycerides, mmol/L^c^	2.4 (1.3)	1.7 (1.0)	1.7 (1.0)
HbA1c, %	5.8 (0.4)	5.3 (0.3)	5.3 (0.3)
**Previously diagnosed coronary heart disease, %**	1.8	0.6	0.7
**Parental history of diabetes, %**	38	18	19
**Mean fasting time (SD), hours**	4.0 (2.6)	3.7 (2.4)	3.7 (2.4)

Participants with missing data: total cholesterol, *n* = 7; LDL cholesterol *n* = 83

*BMI* body mass index, *DBP* diastolic blood pressure, *HC* hip circumference, *HDL* high-density lipoprotein, *LDL* low-density lipoprotein, *SBP* systolic blood pressure, *WC* waist circumference, *WHR* waist-to-hip ratio

^a^Standardised to age and sex structure of the study population

^b^Standardised Townsend Deprivation Index; higher scores represent higher levels of deprivation

^c^Clinical chemistry derived concentrations

**Fig. 1:**
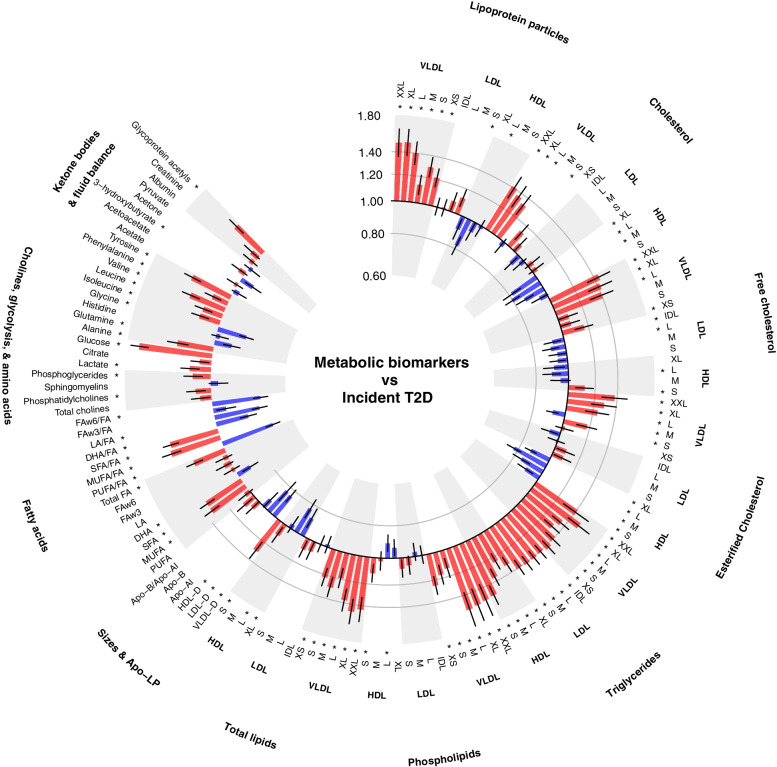
Associations of metabolic biomarkers with risk of incident type 2 diabetes among 50,519 participants in the association analyses population. Hazard ratios (with 95% confidence intervals) are presented per 1−SD higher metabolic biomarker on the natural log scale, stratified by age-at-risk and sex and adjusted for assessment centre, Townsend Deprivation Index, ethnicity, parental history of diabetes, smoking, alcohol drinking, physical activity, dietary factors (whole and refined grains, fruit, vegetables, cheese, unprocessed red meat, processed meat, non-oily and oily fish, type of spread, caffeinated and decaffeinated coffee, tea and dietary supplements), body mass index, waist-to-hip ratio, fasting duration and spectrometer. *False discovery rate controlled *p* < 0.01. Apo-A1, apolipoprotein A1; Apo-B, apolipoprotein B; DHA, docosahexaenoic acid; FA, fatty acids; FAw3, omega-3 fatty acids; FAw6, omega-6 fatty acids; HDL, high-density lipoproteins; HDL-D, high-density lipoprotein particle diameter; IDL, intermediate-density lipoproteins; L, large; LA, linoleic acid; LDL, low-density lipoproteins; LDL-D, low-density lipoprotein particle diameter; LP, lipoprotein; M, medium; MUFA, monounsaturated fatty acids; PUFA, polyunsaturated fatty acids; S, small; SFA, saturated fatty acids; T2D, type 2 diabetes; VLDL, very low-density lipoproteins; VLDL-D, very low-density lipoprotein particle diameter; XL, very large; XS, very small; XXL, extremely large

Among 50,519 participants in the association analyses population, of whom 1211 developed incident T2D (Additional file 1: Table S4), after adjustment for potential confounding factors and accounting for multiple testing, 90 of the 143 metabolic biomarkers showed statistically significant associations with the risk of incident T2D at FDR controlled *p* < 0.01 (Fig. 1, Additional file 1: Table S1, Additional file 1: Fig. S3). Among the strongest positive associations were those of VLDL particle concentrations, particularly larger VLDL particles, and the lipid concentrations within them. Triglyceride concentrations in all 14 lipoprotein subclasses were also very strongly positively associated with incident T2D. Conversely, concentrations of larger HDL particles, and the cholesterol and phospholipids within those particles, were inversely associated with T2D. Higher branched-chain amino acid (BCAA)—leucine, isoleucine and valine—concentrations were associated with a higher risk of T2D, as were higher concentrations of alanine, phenylalanine and tyrosine. Glutamine and glycine were inversely associated with T2D. Relative to total fatty acids, higher concentrations of polyunsaturated, omega-3 and omega-6 fatty acids and docosahexaenoic and linoleic acids were associated with lower T2D risk, whereas higher concentrations of saturated and monounsaturated fatty acids were associated with higher T2D risk. Higher plasma glycoprotein acetyls, a marker of inflammation, were also associated with higher T2D risk.

After additional adjustment for HbA1c, many associations were moderately attenuated but statistically significant associations of most biomarkers (*n* = 76 at FDR controlled *p* < 0.01) with T2D remained (Additional file 1: Table S1). There were no marked differences in the relationships between men and women (Additional file 1: Fig. S4), by age at baseline (Additional file 1: Fig. S5), or after exclusion of the first 3 years of follow-up (Additional file 1: Fig. S6).

The first 11 PCs of the NMR biomarkers explained 90% of the total variance present in the 143 individual biomarkers (Additional file 1: Fig. S7). The PC loadings from these 11 PCs are shown in Fig. S8 (Additional file 1: Fig. S8) (the larger a biomarker’s loading, positive or negative, the more it contributes to that PC), and the associations of these PCs with incident T2D are shown in Table S5 (Additional file 1: Table S5). The major contributors to PC1 were VLDL and LDL particle concentrations and the lipid concentrations within those particles, while for PC2, they included large HDL particles and lipid concentrations within them. PC1 and PC2 showed opposing associations with T2D (adjusted HR 1.25 [95% CI 1.17–1.34] and 0.81 [0.76–0.87], respectively). Biomarkers across multiple molecular pathways, including lipid concentrations in LDL and HDL particles and apolipoprotein concentrations, were prominent contributors to PC3 (HR 1.23 [95% CI 1.17–1.30]). Within PC4 (HR 1.13 [95% CI 1.06–1.20]), loadings were high for small and very large HDL particles and their lipid concentrations, and amino acids were the major contributors to PC5 (1.07 [1.00–1.14]). Fatty acids were dominant in PC6 (0.95 [95% CI 0.89–1.01]) and also PC7 (0.72 [0.67–0.76]), in which ketone bodies also had large factor loadings. Overall, 8 of the 11 PCs were associated with incident T2D independent of sociodemographic characteristics, parental history of diabetes, lifestyle factors, anthropometric measures and fasting time and largely remained so after sequential adjustment for preceding PCs.

In the two traditional risk prediction models, all risk factors were strongly and independently associated with T2D risk among 65,684 participants in the risk prediction population (Additional file 1: Table S6). Older age, male sex, parental history of diabetes, higher levels of adiposity, blood pressure, HbA1c and triglycerides and lower HDL cholesterol concentration were all associated with higher risk. These relationships largely persisted, although with modest attenuation of some, when metabolic biomarker PCs were added. For both models, 8 of the 11 PCs were significantly associated with T2D risk independently of all other risk factors.

**Fig. 2:**
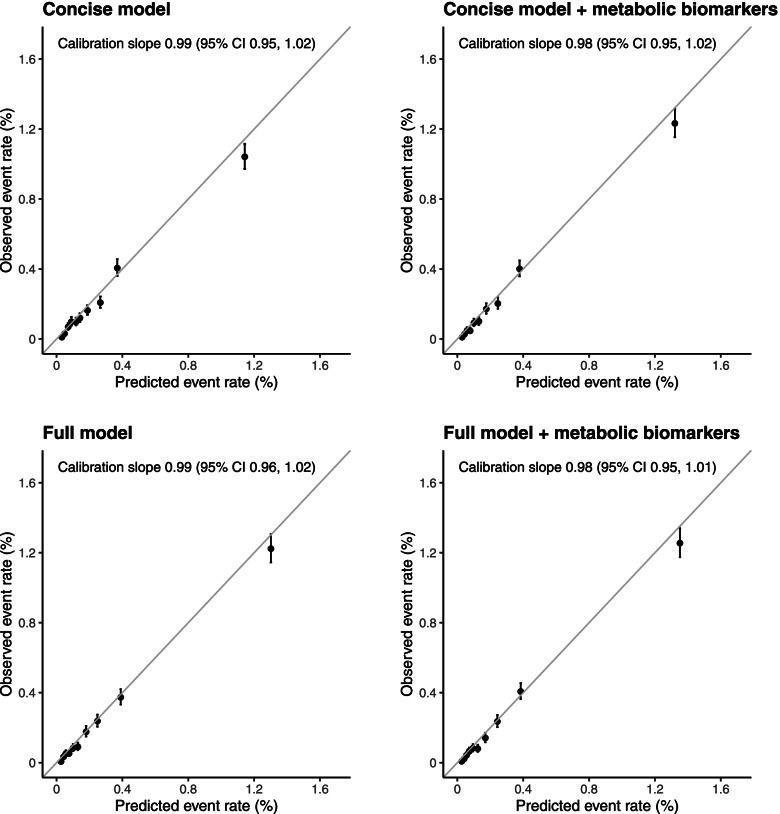
Calibration of risk prediction models for incident type 2 diabetes from cross-validation among 65,684 participants in the risk prediction population. For each model, the observed and predicted T2D event rates are shown for each of 10 equally sized groups of absolute predicted risk. Vertical lines represent 95% CIs. Calibration slopes are presented from 10-fold cross-validation (pooled using inverse variance weighting) and were derived from a Cox regression of the predicted risk on the observed risk. Concise model: age, sex, parental history of diabetes, body mass index and HbA1c. Full model: concise model plus waist circumference, triglycerides and HDL cholesterol. Metabolic biomarkers comprise the first 11 metabolic biomarker principal components

The concise T2D risk prediction model (based on the Framingham ‘personal’ model for T2D risk prediction and incorporating age, sex, parental history of diabetes, BMI and HbA1c) demonstrated good calibration of observed versus predicted T2D rates across deciles of predicted risk (calibration slope, 0.99 [95% CI 0.95–1.02]) (Fig. 2). This did not meaningfully change after the addition of metabolic biomarker PCs (0.98 [95% CI 0.95–1.02]). Table 2 summarises the measures of model fit and performance. The addition of the PCs to the concise model resulted in a 17% increase in the chi-square statistic and yielded an increase in the c-statistic from 0.802 (95% CI 0.791–0.812) to 0.830 (0.822–0.841). Improved T2D risk prediction on addition of the PCs was also evidenced by estimates of the overall continuous NRI (0.44 [95% CI 0.38–0.49]), with an improvement of 0.15 (0.12–0.20) in events and 0.28 (0.26–0.31) in non-events and both absolute (1.5 [1.0–1.9]) and relative (15.0% [10.5–20.4%]) IDI. The full model (concise model plus blood pressure, WC, HDL cholesterol and triglycerides, based on the Framingham ‘clinical’ model for T2D risk prediction) achieved a c-statistic of 0.829 (95% CI 0.819–0.838). Modest improvements in model fit and performance were observed following the addition of metabolic biomarker PCs to this model, with a 6% increase in the chi-square statistic, a c-statistic of 0.837 (95% CI 0.831–0.848), an overall continuous NRI of 0.22 (0.17–0.28), an absolute IDI of 0.7 (0.4–1.1) and a relative IDI of 6.3% (4.1–9.8%). The full model was well-calibrated, both with and without the inclusion of metabolic biomarker PCs (0.99 [95% CI 0.96–1.02] and 0.98 [0.95–1.01], respectively). When analyses were repeated among participants taking lipid-lowering medications at baseline, c-statistics for the four individual T2D risk prediction models were lower than in the main study population, but estimates of the relative performance of the nested models were broadly comparable (Additional file 1: Table S7). Sensitivity analyses replacing WC in the full model with WHR did not materially affect the performance of the model (Additional file 1: Table S8). Including covariates as continuous variables improved the discriminatory ability of both the concise and the full model; although moderately diminished, the ability of metabolic biomarker PCs to improve T2D risk prediction remained (Additional file 1: Table S9).

**Table 2:** Performance of risk prediction models for incident type 2 diabetes among 65,684 participants in the risk prediction population

Performance metric	Concise model^a^	Concise model^a^ plus metabolic biomarkers^b^	Full model^c^	Full model^c^ plus metabolic biomarkers^b^
**C-statistic (CI)**^d^	0.802 (0.791, 0.812)	0.830 (0.822, 0.841)	0.829 (0.819, 0.838)	0.837 (0.831, 0.848)
**Metrics of relative performance**				
*χ*^2 e,f^	453 (*p* < 0.0001)		177 (*p* < 0.0001)	
%increase *χ*^2^	17		6	
Absolute IDI ^f,g^	1.5 (1.0, 1.9)		0.7 (0.4, 1.1)	
Relative IDI (%) (CI) ^f,g^	15.0 (10.5, 20.4)		6.3 (4.1, 9.8)	
Continuous NRI (CI)^f,h^				
Events	0.15 (0.12, 0.20)		0.10 (0.06, 0.14)	
Non-events	0.28 (0.26, 0.31)		0.12 (0.09, 0.14)	
Overall	0.44 (0.38, 0.49)		0.22 (0.17, 0.28)	

*DF* degrees of freedom, *IDI* integrated discrimination improvement, *NRI* net reclassification improvement; *T2D* type 2 diabetes

^a^Concise model: age, sex, parental history of diabetes, body mass index and HbA1c

^b^Metabolic biomarkers comprise the first 11 metabolic biomarker principal components

^c^Full model: concise model plus waist circumference, blood pressure, triglycerides and HDL cholesterol

^d^The c-statistic measures the ability of a model to rank participants from low to high risk. Given two randomly selected individuals, one who develops T2D and one who does not, the c-statistic is the probability that the model will give a higher predicted risk for the individual who develops T2D. An uninformative model will have a c-statistic of 0.5 and a model that discriminates perfectly will have a c-statistic of 1.0

^e^11 DF

^f^Bias-corrected estimates and confidence intervals were derived using 200 bootstrap samples

^g^The IDI quantifies the difference between two models in their ability to predict risk. It is calculated as the difference between the two models in the mean predicted T2D risk among those who did develop T2D minus the mean predicted risk of T2D in those who did not develop T2D (i.e. it is the difference between two differences). When metabolic biomarkers were added to the concise model, the separation in the mean predicted T2D risk between those who did develop T2D, compared with those who did not develop T2D, increased in relative terms by 15.0%. Positive IDI values indicate improved T2D risk classification following the addition of metabolic biomarkers to the risk prediction model

^h^The continuous NRI quantifies the appropriateness of the change in predicted probabilities of T2D between two models. The ‘events’ NRI is calculated among those who developed T2D, and the ‘non-events’ NRI is calculated among those who did not develop T2D. Both statistics are calculated as the probability of an ‘appropriate’ change in predicted risk (after the addition of metabolic biomarkers to the model) minus the probability of an ‘inappropriate’ change in predicted risk. For those who developed T2D, an appropriate change would be a higher predicted T2D risk after the addition of metabolic biomarkers to the model. An inappropriate change would be a lower predicted T2D risk after the addition of metabolic biomarkers to the model. When metabolic biomarkers were added to the concise model, among those who developed T2D, 15% more were assigned a higher predicted T2D risk than were assigned a lower predicted risk. The overall NRI is the sum of the ‘events’ and ‘non-events’ NRI statistics. Positive NRI values indicate that the addition of metabolic biomarkers results in a superior model

## Discussion

This prospective population-based cohort study of over 65,000 middle-aged adults with 1719 cases of new-onset T2D is, to our knowledge, the largest study to date to examine the predictive value of circulating metabolic biomarkers for T2D risk. Strong independent associations of diverse biomarkers, quantified using targeted NMR-based metabolomic profiling, including lipoprotein particle size and composition, amino acids and fatty acids, with risk of incident T2D were observed. When added to established risk prediction models, PCs derived from 143 circulating biomarkers achieved modest improvements in T2D risk prediction.

Our study found strong positive associations of VLDL particle measures and triglyceride concentrations with incident T2D risk and inverse associations of HDL particle size and lipids within larger HDL particles. These findings are qualitatively, and broadly quantitatively, consistent with previous studies [29, 30], and are characteristic of lipoprotein profiles associated with insulin resistance [31]. This is also thought to underlie the strong positive associations of BCAAs—leucine, isoleucine and valine—with the risk of T2D observed in UKB and in previous studies among diverse populations [9, 11, 29]. More specifically, genetic association studies have shown increased BCAA levels as a consequence of insulin resistance [32], which, in turn, appear to be causally related to T2D [33]. We replicated the findings of studies showing higher levels of phenylalanine, tyrosine and alanine [9, 11, 29], and lower concentrations of glutamate [9, 29] and glycine [9] several years prior to T2D diagnosis, and the observed T2D-associated fatty acid profiles are broadly consistent with previous investigations [29, 34]. Insulin resistance and inflammation are postulated to underlie some or all of these associations [9, 11, 34], but the nature of the relationships of these and other metabolic biomarkers with T2D, including their causal significance, remains uncertain. Despite this, these findings provide clear evidence of the relevance of diverse metabolic biomarkers to T2D risk.

The ‘traditional’ T2D risk prediction models examined in the present study, based on the Framingham ‘personal’ and ‘clinical’ diabetes risk scores, demonstrated good discriminatory ability in the UKB population (concise model: c-statistic 0.80; full model: c-statistic 0.83). This is consistent with the performance of similar models across varied populations [35], highlighting one of the major challenges of identifying novel predictive biomarkers for T2D. That is, that established clinical risk factors perform so well in predicting T2D risk that achieving clinically meaningful improvements above and beyond these is difficult.

The addition of metabolic biomarkers to the concise model in the present study improved, albeit modestly, model fit and risk discrimination (c-statistic 0.83). Although some previous studies have observed no improvement in risk discrimination with the addition of metabolic biomarkers to similar traditional risk prediction models [12, 15, 36], several have investigated the impact of only limited biomarkers [12, 15]. The inclusion of more diverse biomarkers has tended to achieve greater gains in model discrimination [10, 17, 29]. For example, in a case-cohort study in Germany, comprising 800 T2D cases and a randomly selected subcohort of 2282 adults (mean follow-up of 7 years), the addition of 14 metabolic biomarkers (including hexoses, amino acids and fatty acids) to an established T2D risk score, comprising clinical risk factors and glycaemia, resulted in moderate, but statistically significant, improvement in risk discrimination (increase in c-statistic from 0.901 to 0.912; *p* < 0.0001) [10]. However, even these studies have tended to investigate highly selected subsets of biomarkers. In contrast, the use of principal component analysis in the present study facilitated the inclusion of information from all 143 metabolic biomarkers, despite their highly correlated nature. Many individual biomarkers most strongly associated with T2D were prominent contributors to the PCs selected for inclusion in risk prediction models, most of which were associated with incident T2D in fully adjusted regression models. The more modest, and non-significant, gains in risk discrimination when metabolic biomarkers were added to the full model may reflect the insensitivity of the c-statistic to improvements in predictive performance with the addition of new, even strong, risk predictors to established models [37]. It likely also reflects an overlap between measured metabolic biomarkers and blood-based risk factors included in this model. These findings suggest that there may be limited value for T2D risk discrimination of adding metabolic biomarkers to a risk prediction model which already includes routine clinical chemistry lipid measures. However, the inclusion of metabolic biomarkers instead of these routine lipid measures would be expected to enhance T2D risk prediction model performance. Moreover, metabolomic profiling data may be of wider clinical relevance (e.g. for diagnosis and risk assessment of other cardiometabolic diseases) [23].

More global measures of model performance provided supportive evidence of the value of metabolic biomarkers for T2D risk prediction. Their addition to both traditional risk prediction models in the present study was associated with improvement in the prediction of T2D using measures of risk reclassification, specifically the IDI and continuous NRI. Of note, the NRI appeared to be driven more by reductions in predicted risk among participants who did not develop T2D, suggesting metabolomic profiling may be particularly valuable for reducing unnecessary prevention interventions among individuals at low risk of T2D. Increasing availability of standardised, quantitative, high-throughput metabolomics platforms, such as that used in the current study, underscores the potential translational relevance of these findings.

In addition to a large number of incident T2D events, our study has several strengths. An established targeted NMR metabolomics platform, with existing clinical regulatory approvals [24], was used; as well as enabling quantification of diverse biomarkers, this facilitates comparisons between study populations and enhances the potential clinical relevance. Moreover, high levels of correlation between NMR- and standard clinical chemistry-derived concentrations of a subset of biomarkers (Additional file 1: Fig. S9) support the validity of the approach [38]. The exclusion of participants taking lipid-lowering medication avoided treatment-associated biases, although the broadly comparable performance of the nested risk prediction models in this subpopulation (with a higher frequency of incident T2D) demonstrates the wider generalisability of our findings. Finally, the cohort study design avoided potential biases and loss of precision which may affect more frequently used nested case-control and case-cohort designs. However, the study also has limitations. Incident T2D was limited to diagnosed cases; although resulting misclassification would likely underestimate associations of metabolic biomarkers with T2D, the relative improvements in model performance (between models with versus without metabolic biomarkers) should be largely unaffected by misclassification in outcome assessment. Blood samples were taken in the non-fasting state, and so would be subject to greater variability in metabolic biomarker concentrations than fasting samples (although fasting duration has previously been found to account for only a small proportion of variation in plasma metabolic biomarker concentrations [39]). However, our analyses were adjusted for fasting time, as well as for extensive dietary factors, which should have limited any material impact of the use of non-fasting samples on the findings. Moreover, although risk prediction would ideally incorporate repeat biomarker levels measured longitudinally, the use of single measurements more closely reflects the practical implementation of risk prediction models in the clinical setting. Independent validation of the risk prediction findings was not performed. However, the observed associations of metabolic biomarkers with T2D replicate previous study findings [9, 11, 29, 30, 34], with no novel associations identified. Finally, the more favourable lifestyle and health-related characteristics of the UKB population when compared with the general UK population (e.g. participants were less likely to be obese or to smoke and had fewer chronic diseases at recruitment) [40] would not be expected to impact on the generalisability of observed associations of metabolic biomarkers with T2D risk [40, 41]. However, the risk prediction findings may not necessarily be generalisable to other populations at higher risk of future T2D.

## Conclusions

In summary, this study provides large-scale evidence of the incremental predictive value of metabolomic profiling for the prediction of T2D risk. Addition of data on 143 circulating metabolic biomarkers, with replicated prospective associations with T2D, to an established risk prediction model comprising basic clinical risk factors and HbA1c improved T2D risk discrimination and classification. More modest improvements were observed when metabolic biomarkers were added to a model additionally incorporating WC, blood pressure, and plasma lipid measures. The study serves to illustrate the utility of large-scale biobanks for the assessment of the clinical relevance and value of emerging biomarkers. Moreover, given increasing availability, including in clinical settings, of high-throughput, comprehensive, targeted metabolomic profiling, these findings may have translational relevance for T2D risk stratification and precision prevention.

## Supplementary Information

**Additional file 1: **Predictive value of circulating NMR metabolic biomarkers for type 2 diabetes risk in the UK Biobank study. **Table S1.** Distribution of metabolic biomarkers and their associations with incident type 2 diabetes among 50,519 participants in the association analyses population. **Table S2.** Diagnosis and medication codes for assessment of type 2 diabetes status in primary and secondary healthcare and death registry records and UK Biobank verbal interview. **Table S3.** Baseline characteristics of participants with and without NMR-metabolomics profiling. **Table S4.** Baseline characteristics of 50,519 participants in the association analyses population by incident type 2 diabetes status. **Table S5.** Associations of the first 11 metabolic biomarker principal components with risk of incident type 2 diabetes among 50,519 participants in the association analyses population. **Table S6.** Regression models for risk of incident type 2 diabetes among 65,684 participants in the risk prediction population. **Table S7.** Performance of risk prediction models for incident type 2 diabetes among 13,695 participants taking lipid-lowering medication at recruitment. **Table S8.** Performance of risk prediction models including waist-to-hip ratio for incident type 2 diabetes among 65,684 participants in the risk prediction population. **Table S9.** Performance of risk prediction models for incident type 2 diabetes incorporating co-variates, where relevant, as continuous variables among 65,684 participants in the risk prediction population. **Fig. S1.** Participant exclusions to derive risk prediction and association analyses populations. **Fig. S2.** Cross-correlations of metabolic biomarkers. **Fig. S3.** Associations of metabolic biomarkers with risk of incident type 2 diabetes among 50,519 participants in the association analyses population. **Fig. S4.** Associations of metabolic biomarkers with risk of incident type 2 diabetes by sex among 50,519 participants in the association analyses population. **Fig. S5.** Associations of metabolic biomarkers with risk of incident type 2 diabetes by age among 50,519 participants in the association analyses population. **Fig. S6.** Associations of metabolic biomarkers with risk of incident type 2 diabetes excluding the first three years of follow−up in the association analyses population. **Fig. S7.** Importance of the first 20 metabolic biomarker principal components. **Fig. S8.** Characterisation of the first 11 metabolic biomarker principal components among 65,684 participants in the risk prediction population. **Fig. S9.** Comparison of biomarkers measured by NMR and routine clinical chemistry assays among 65,684 participants in the risk prediction population.

## Data Availability

The underlying data are open access through application to the UK Biobank, and materials and methods will be made freely available through the UK Biobank as part of this project.

## Abbreviations

BCAA: Branched-chain amino acid
BMI: Body mass index
CI: Confidence interval
DBP: Diastolic blood pressure
FDR: False discovery rate
HC: Hip circumference
HDL: High-density lipoprotein
IDI: Integrated discrimination index
IQR: Interquartile range
LDL: Low-density lipoprotein
NMR: Nuclear magnetic resonance
NRI: Net reclassification improvement
PC: Principal component
SBP: Systolic blood pressure
SD: Standard deviation
T2D: Type 2 diabetes
UKB: UK Biobank
VLDL: Very low-density lipoprotein
WC: Waist circumference
WHR: Waist-to-hip ratio
