# Supplementary material for: Predictive value of circulating NMR metabolic biomarkers for type 2 diabetes risk in the UK Biobank study
Source: BMC Med. 2022 May 3;20:159. doi: 10.1186/s12916-022-02354-9 (PMC9063288; doi:10.1186/s12916-022-02354-9)
Supplement: Supplementary file 1 — Additional file 1: Predictive value of circulating NMR metabolic biomarkers for type 2 diabetes risk in the UK Biobank study. Table S1. Distribution of metabolic biomarkers and their associations with incident type 2 diabetes among 50,519 participants in the association analyses population. Table S2. Diagnosis and medication codes for assessment of type 2 diabetes status in primary and secondary healthcare and death registry records and UK Biobank verbal interview. Table S3. Baseline characteristics of participants with and without NMR-metabolomics profiling. Table S4. Baseline characteristics of 50,519 participants in the association analyses population by incident type 2 diabetes status. Table S5. Associations of the first 11 metabolic biomarker principal components with risk of incident type 2 diabetes among 50,519 participants in the association analyses population. Table S6. Regression models for risk of incident type 2 diabetes among 65,684 participants in the risk prediction population. Table S7. Performance of risk prediction models for incident type 2 diabetes among 13,695 participants taking lipid-lowering medication at recruitment. Table S8. Performance of risk prediction models including waist-to-hip ratio for incident type 2 diabetes among 65,684 participants in the risk prediction population. Table S9. Performance of risk prediction models for incident type 2 diabetes incorporating co-variates, where relevant, as continuous variables among 65,684 participants in the risk prediction population. Fig. S1. Participant exclusions to derive risk prediction and association analyses populations. Fig. S2. Cross-correlations of metabolic biomarkers. Fig. S3. Associations of metabolic biomarkers with risk of incident type 2 diabetes among 50,519 participants in the association analyses population. Fig. S4. Associations of metabolic biomarkers with risk of incident type 2 diabetes by sex among 50,519 participants in the association analyses population. Fig. S5. As [file 12916_2022_2354_MOESM1_ESM.pdf]

This page is intentionally left blank

Predictive value of circulating NMR metabolic biomarkers for type 2 diabetes risk in the UK Biobank study

Additional File 1, Table of Contents

Table S1	Distribution of metabolic biomarkers and their associations with incident type 2 diabetes among 50,519 participants in the association analyses population	Page 2
Table S2	Diagnosis and medication codes for assessment of type 2 diabetes status in primary and secondary healthcare and death registry records and UK Biobank verbal interview	Page 8
Table S3	Baseline characteristics of participants with and without NMR-metabolomics profiling	Page 10
Table S4	Baseline characteristics of 50,519 participants in the association analyses population by incident type 2 diabetes status	Page 11
Table S5	Associations of the first 11 metabolic biomarker principal components with risk of incident type 2 diabetes among 50,519 participants in the association analyses population	Page 12
Table S6	Regression models for risk of incident type 2 diabetes among 65,684 participants in the risk prediction population	Page 13
Table S7	Performance of risk prediction models for incident type 2 diabetes among 13,695 participants taking lipid-lowering medication at recruitment	Page 14
Table S8	Performance of risk prediction models including waist-to-hip ratio for incident type 2 diabetes among 65,684 participants in the risk prediction population	Page 15
Table S9	Performance of risk prediction models for incident type 2 diabetes incorporating co-variates, where relevant, as continuous variables among 65,684 participants in the risk prediction population	Page 16
Figure S1	Participant exclusions to derive risk prediction and association analyses populations	Page 17
Figure S2	Cross-correlations of metabolic biomarkers	Page 18
Figure S3	Associations of metabolic biomarkers with risk of incident type 2 diabetes among 50,519 participants in the association analyses population	Page 19
Figure S4	Associations of metabolic biomarkers with risk of incident type 2 diabetes by sex among 50,519 participants in the association analyses population	Page 26
Figure S5	Associations of metabolic biomarkers with risk of incident type 2 diabetes by age among 50,519 participants in the association analyses population	Page 27
Figure S6	Associations of metabolic biomarkers with risk of incident type 2 diabetes excluding the first three years of follow−up in the association analyses population	Page 28
Figure S7	Importance of the first 20 metabolic biomarker principal components	Page 29
Figure S8	Characterisation of the first 11 metabolic biomarker principal components among 65,684 participants in the risk prediction population	Page 30
Figure S9	Comparison of biomarkers measured by NMR and routine clinical chemistry assays among 65,684 participants in the risk prediction population	Page 32

Table S1. Distribution of metabolic biomarkers and their associations with incident type 2 diabetes among 50,519 participants in the association analyses population

Metabolic biomarker	Mean (SD)	Hazard ratio (95% CI) per 1-SD higher level on the natural log scale					
		Main model*		Main model + HbA1c		Main model excluding first 3 years of follow-up	
		HR (95% CI) †	p-value §	HR (95% CI) †	p-value §	HR (95% CI) †	p-value §
**Lipoprotein particle concentrations**							
Chylomicrons and extremely large VLDL particles	1.7^{-06} (1.5^{-06}) mmol/L	1.49 (1.40, 1.58)	3.6^{-16}	1.32 (1.22, 1.41)	2.2^{-08}	1.47 (1.37, 1.58)	3.6^{-13}
Very large VLDL particles	3.4^{-06} (2.2^{-06}) mmol/L	1.50 (1.41, 1.59)	6.7^{-18}	1.30 (1.21, 1.39)	4.4^{-08}	1.47 (1.37, 1.57)	5.1^{-14}
Large VLDL particles	1.0^{-05} (5.4^{-06}) mmol/L	1.40 (1.32, 1.48)	9.8^{-17}	1.24 (1.16, 1.31)	3.4^{-07}	1.38 (1.30, 1.47)	2.7^{-13}
Medium VLDL particles	3.6^{-05} (1.2^{-05}) mmol/L	1.13 (1.07, 1.20)	4.6^{-04}	1.03 (0.97, 1.10)	4.1^{-01}	1.13 (1.06, 1.20)	1.5^{-03}
Small VLDL particles	3.9^{-05} (1.3^{-05}) mmol/L	1.29 (1.23, 1.36)	4.3^{-13}	1.15 (1.09, 1.22)	1.0^{-04}	1.28 (1.21, 1.36)	1.4^{-10}
Very small VLDL particles	5.7^{-05} (1.3^{-05}) mmol/L	1.22 (1.15, 1.28)	3.3^{-09}	1.09 (1.03, 1.16)	9.4^{-03}	1.21 (1.14, 1.28)	1.5^{-07}
IDL particles	3.2^{-04} (7.0^{-05}) mmol/L	1.00 (0.94, 1.07)	8.8^{-01}	0.94 (0.88, 1.00)	8.2^{-02}	1.01 (0.95, 1.08)	7.6^{-01}
Large LDL particles	7.5^{-04} (1.6^{-04}) mmol/L	1.01 (0.95, 1.07)	7.6^{-01}	0.95 (0.89, 1.01)	1.2^{-01}	1.02 (0.95, 1.08)	6.5^{-01}
Medium LDL particles	3.0^{-04} (7.2^{-05}) mmol/L	1.08 (1.01, 1.14)	3.2^{-02}	0.99 (0.93, 1.05)	7.8^{-01}	1.08 (1.01, 1.15)	3.3^{-02}
Small LDL particles	1.7^{-04} (3.6^{-05}) mmol/L	1.12 (1.06, 1.18)	4.6^{-04}	1.02 (0.96, 1.09)	5.4^{-01}	1.12 (1.06, 1.19)	1.3^{-03}
Very large HDL particles	2.4^{-04} (9.3^{-05}) mmol/L	0.91 (0.83, 0.98)	1.9^{-02}	0.92 (0.85, 1.00)	6.7^{-02}	0.90 (0.82, 0.99)	2.4^{-02}
Large HDL particles	1.5^{-03} (7.6^{-04}) mmol/L	0.82 (0.75, 0.88)	1.1^{-08}	0.87 (0.80, 0.94)	4.1^{-04}	0.81 (0.74, 0.88)	2.8^{-08}
Medium HDL particles	3.8^{-03} (9.0^{-04}) mmol/L	0.93 (0.87, 1.00)	4.4^{-02}	0.98 (0.91, 1.04)	5.4^{-01}	0.92 (0.85, 0.99)	2.8^{-02}
Small HDL particles	9.6^{-03} (1.2^{-03}) mmol/L	0.98 (0.92, 1.04)	5.9^{-01}	0.97 (0.91, 1.04)	4.5^{-01}	0.98 (0.91, 1.05)	6.2^{-01}
**Cholesterol concentrations**							
Chylomicrons and extremely large VLDL particles	0.06 (0.04) mmol/L	1.41 (1.33, 1.50)	7.2^{-15}	1.26 (1.18, 1.35)	2.2^{-07}	1.40 (1.31, 1.49)	4.1^{-12}
Very large VLDL particles	0.05 (0.03) mmol/L	1.36 (1.28, 1.45)	4.9^{-12}	1.20 (1.11, 1.28)	1.1^{-04}	1.35 (1.26, 1.44)	1.0^{-09}
Large VLDL particles	0.10 (0.05) mmol/L	1.32 (1.24, 1.41)	5.0^{-11}	1.17 (1.09, 1.25)	3.9^{-04}	1.31 (1.22, 1.40)	6.7^{-09}
Medium VLDL particles	0.18 (0.06) mmol/L	0.97 (0.91, 1.02)	2.8^{-01}	0.92 (0.86, 0.98)	7.5^{-03}	0.97 (0.91, 1.04)	4.3^{-01}
Small VLDL particles	0.16 (0.05) mmol/L	1.14 (1.07, 1.21)	3.2^{-04}	1.03 (0.96, 1.10)	4.4^{-01}	1.14 (1.07, 1.21)	9.1^{-04}
Very small VLDL particles	0.19 (0.05) mmol/L	1.06 (1.00, 1.13)	7.2^{-02}	0.99 (0.93, 1.05)	7.9^{-01}	1.06 (1.00, 1.13)	9.5^{-02}
IDL particles	0.88 (0.19) mmol/L	0.93 (0.87, 0.99)	1.6^{-02}	0.90 (0.84, 0.96)	7.1^{-04}	0.93 (0.87, 1.00)	4.9^{-02}
Large LDL particles	1.16 (0.26) mmol/L	0.97 (0.91, 1.02)	2.7^{-01}	0.93 (0.87, 0.98)	1.5^{-02}	0.97 (0.91, 1.04)	4.5^{-01}
Medium LDL particles	0.43 (0.11) mmol/L	1.04 (0.98, 1.10)	2.6^{-01}	0.97 (0.91, 1.03)	3.8^{-01}	1.05 (0.98, 1.12)	2.0^{-01}
Small LDL particles	0.19 (0.04) mmol/L	1.03 (0.97, 1.10)	3.1^{-01}	0.96 (0.90, 1.02)	3.0^{-01}	1.04 (0.98, 1.11)	2.5^{-01}
Very large HDL particles	0.09 (0.03) mmol/L	0.82 (0.74, 0.89)	5.1^{-08}	0.84 (0.77, 0.91)	1.5^{-05}	0.82 (0.74, 0.89)	7.7^{-07}

Table S1. Distribution of metabolic biomarkers and their associations with incident type 2 diabetes among 50,519 participants in the association analyses population

Metabolic biomarker	Mean (SD)	Hazard ratio (95% CI) per 1-SD higher level on the natural log scale					
		Main model*		Main model + HbA1c		Main model excluding first 3 years of follow-up	
		HR (95% CI) †	p-value §	HR (95% CI) †	p-value §	HR (95% CI) †	p-value §
Large HDL particles	0.31 (0.17) mmol/L	0.80 (0.74, 0.85)	7.0^{-15}	0.85 (0.79, 0.91)	5.5^{-07}	0.80 (0.74, 0.86)	2.7^{-13}
Medium HDL particles	0.49 (0.12) mmol/L	0.86 (0.80, 0.93)	1.7^{-05}	0.92 (0.85, 0.98)	2.1^{-02}	0.85 (0.78, 0.92)	3.0^{-05}
Small HDL particles	0.44 (0.06) mmol/L	0.94 (0.88, 1.01)	8.9^{-02}	0.94 (0.88, 1.01)	1.1^{-01}	0.95 (0.88, 1.01)	1.2^{-01}
**Free cholesterol concentrations**							
Chylomicrons and extremely large VLDL particles	0.02 (0.02) mmol/L	1.45 (1.36, 1.53)	6.3^{-17}	1.30 (1.21, 1.38)	6.1^{-09}	1.43 (1.34, 1.52)	1.1^{-13}
Very large VLDL particles	0.02 (0.01) mmol/L	1.42 (1.34, 1.51)	6.3^{-15}	1.24 (1.16, 1.33)	1.8^{-06}	1.40 (1.31, 1.49)	6.7^{-12}
Large VLDL particles	0.05 (0.02) mmol/L	1.40 (1.32, 1.48)	1.2^{-14}	1.23 (1.14, 1.31)	5.0^{-06}	1.38 (1.29, 1.47)	9.5^{-12}
Medium VLDL particles	0.08 (0.03) mmol/L	1.08 (1.01, 1.14)	3.8^{-02}	0.99 (0.93, 1.06)	8.3^{-01}	1.08 (1.01, 1.15)	5.4^{-02}
Small VLDL particles	0.06 (0.02) mmol/L	1.07 (1.01, 1.14)	3.7^{-02}	0.99 (0.92, 1.05)	7.7^{-01}	1.08 (1.01, 1.15)	4.9^{-02}
Very small VLDL particles	0.06 (0.01) mmol/L	1.16 (1.10, 1.23)	5.3^{-06}	1.06 (0.99, 1.12)	1.1^{-01}	1.16 (1.09, 1.23)	4.5^{-05}
IDL particles	0.23 (0.05) mmol/L	0.92 (0.86, 0.98)	9.5^{-03}	0.89 (0.83, 0.95)	1.5^{-04}	0.93 (0.87, 1.00)	4.1^{-02}
Large LDL particles	0.31 (0.07) mmol/L	0.90 (0.85, 0.96)	5.7^{-04}	0.88 (0.83, 0.94)	4.2^{-05}	0.91 (0.85, 0.97)	6.3^{-03}
Medium LDL particles	0.12 (0.03) mmol/L	0.94 (0.88, 0.99)	3.7^{-02}	0.90 (0.85, 0.96)	9.8^{-04}	0.95 (0.89, 1.01)	1.2^{-01}
Small LDL particles	0.05 (0.01) mmol/L	0.93 (0.88, 0.99)	1.6^{-02}	0.91 (0.86, 0.96)	4.8^{-04}	0.94 (0.88, 1.00)	5.4^{-02}
Very large HDL particles	0.02 (0.01) mmol/L	0.91 (0.84, 0.98)	1.3^{-02}	0.91 (0.84, 0.98)	2.2^{-02}	0.91 (0.83, 0.99)	2.9^{-02}
Large HDL particles	0.07 (0.04) mmol/L	0.89 (0.83, 0.95)	3.9^{-04}	0.92 (0.86, 0.99)	3.4^{-02}	0.89 (0.82, 0.95)	4.8^{-04}
Medium HDL particles	0.09 (0.02) mmol/L	0.95 (0.88, 1.01)	1.3^{-01}	0.98 (0.91, 1.05)	6.1^{-01}	0.93 (0.86, 1.01)	7.7^{-02}
Small HDL particles	0.11 (0.02) mmol/L	1.12 (1.06, 1.18)	7.6^{-04}	1.07 (1.01, 1.13)	4.7^{-02}	1.11 (1.04, 1.17)	5.9^{-03}
**Esterified cholesterol concentrations**							
Chylomicrons and extremely large VLDL particles	0.03 (0.02) mmol/L	1.38 (1.29, 1.46)	1.2^{-12}	1.23 (1.15, 1.32)	5.7^{-06}	1.37 (1.27, 1.46)	2.2^{-10}
Very large VLDL particles	0.03 (0.01) mmol/L	1.29 (1.20, 1.37)	2.3^{-08}	1.13 (1.05, 1.22)	7.4^{-03}	1.28 (1.19, 1.37)	6.3^{-07}
Large VLDL particles	0.05 (0.02) mmol/L	1.24 (1.16, 1.32)	4.0^{-07}	1.11 (1.03, 1.19)	2.0^{-02}	1.23 (1.14, 1.32)	7.3^{-06}
Medium VLDL particles	0.10 (0.03) mmol/L	0.92 (0.87, 0.96)	6.0^{-05}	0.90 (0.85, 0.94)	1.6^{-06}	0.92 (0.87, 0.96)	5.7^{-04}
Small VLDL particles	0.10 (0.03) mmol/L	1.18 (1.11, 1.25)	1.1^{-05}	1.06 (0.99, 1.13)	1.5^{-01}	1.18 (1.10, 1.25)	5.6^{-05}
Very small VLDL particles	0.13 (0.03) mmol/L	1.02 (0.96, 1.08)	5.9^{-01}	0.96 (0.90, 1.02)	2.6^{-01}	1.02 (0.95, 1.09)	5.8^{-01}
IDL particles	0.65 (0.14) mmol/L	0.93 (0.87, 0.99)	2.0^{-02}	0.90 (0.85, 0.96)	1.3^{-03}	0.94 (0.87, 1.00)	5.3^{-02}
Large LDL particles	0.85 (0.19) mmol/L	0.99 (0.93, 1.05)	7.6^{-01}	0.94 (0.88, 1.00)	6.9^{-02}	1.00 (0.93, 1.06)	9.4^{-01}

Table S1. Distribution of metabolic biomarkers and their associations with incident type 2 diabetes among 50,519 participants in the association analyses population

Metabolic biomarker	Mean (SD)	Hazard ratio (95% CI) per 1-SD higher level on the natural log scale					
		Main model*		Main model + HbA1c		Main model excluding first 3 years of follow-up	
		HR (95% CI) †	p-value §	HR (95% CI) †	p-value §	HR (95% CI) †	p-value §
Medium LDL particles	0.31 (0.08) mmol/L	1.08 (1.02, 1.15)	2.3^{-02}	1.00 (0.93, 1.06)	9.2^{-01}	1.09 (1.02, 1.16)	2.5^{-02}
Small LDL particles	0.13 (0.03) mmol/L	1.08 (1.02, 1.14)	2.3^{-02}	1.00 (0.93, 1.06)	9.2^{-01}	1.09 (1.02, 1.15)	2.4^{-02}
Very large HDL particles	0.06 (0.03) mmol/L	0.87 (0.83, 0.91)	5.0^{-11}	0.86 (0.81, 0.91)	7.6^{-08}	0.87 (0.82, 0.91)	1.9^{-09}
Large HDL particles	0.24 (0.13) mmol/L	0.78 (0.72, 0.83)	1.7^{-19}	0.83 (0.77, 0.89)	6.1^{-09}	0.78 (0.73, 0.84)	5.0^{-17}
Medium HDL particles	0.41 (0.10) mmol/L	0.84 (0.78, 0.91)	6.8^{-07}	0.90 (0.84, 0.97)	6.2^{-03}	0.84 (0.77, 0.91)	2.0^{-06}
Small HDL particles	0.33 (0.05) mmol/L	0.89 (0.83, 0.95)	5.5^{-04}	0.91 (0.84, 0.97)	4.6^{-03}	0.90 (0.83, 0.96)	2.8^{-03}
**Triglyceride concentrations**							
Chylomicrons and extremely large VLDL particles	0.13 (0.13) mmol/L	1.41 (1.30, 1.51)	3.3^{-10}	1.24 (1.15, 1.34)	3.1^{-05}	1.42 (1.31, 1.54)	5.2^{-09}
Very large VLDL particles	0.11 (0.08) mmol/L	1.51 (1.42, 1.60)	2.6^{-18}	1.30 (1.22, 1.39)	2.9^{-08}	1.48 (1.38, 1.57)	4.0^{-14}
Large VLDL particles	0.16 (0.09) mmol/L	1.35 (1.28, 1.43)	8.6^{-16}	1.21 (1.14, 1.28)	8.3^{-07}	1.33 (1.26, 1.41)	1.9^{-12}
Medium VLDL particles	0.27 (0.11) mmol/L	1.32 (1.25, 1.38)	1.8^{-14}	1.18 (1.11, 1.25)	5.4^{-06}	1.30 (1.23, 1.37)	1.7^{-11}
Small VLDL particles	0.15 (0.06) mmol/L	1.40 (1.34, 1.47)	3.8^{-22}	1.26 (1.19, 1.32)	2.3^{-10}	1.38 (1.31, 1.45)	4.4^{-17}
Very small VLDL particles	0.07 (0.02) mmol/L	1.43 (1.37, 1.49)	5.8^{-30}	1.28 (1.22, 1.34)	2.4^{-14}	1.41 (1.34, 1.48)	3.1^{-23}
IDL particles	0.10 (0.03) mmol/L	1.41 (1.35, 1.47)	2.3^{-30}	1.27 (1.21, 1.32)	2.7^{-14}	1.39 (1.33, 1.45)	7.9^{-24}
Large LDL particles	0.10 (0.02) mmol/L	1.41 (1.36, 1.47)	2.0^{-31}	1.27 (1.21, 1.32)	2.4^{-14}	1.40 (1.34, 1.46)	6.7^{-25}
Medium LDL particles	0.03 (0.01) mmol/L	1.42 (1.36, 1.47)	2.3^{-31}	1.27 (1.21, 1.32)	2.4^{-14}	1.40 (1.34, 1.46)	7.9^{-25}
Small LDL particles	0.02 (0.01) mmol/L	1.40 (1.34, 1.46)	1.1^{-28}	1.26 (1.20, 1.32)	2.1^{-13}	1.38 (1.32, 1.45)	1.8^{-22}
Very large HDL particles	7.1^{-03} (2.6^{-03}) mmol/L	1.32 (1.26, 1.37)	6.4^{-20}	1.22 (1.16, 1.27)	1.5^{-10}	1.29 (1.23, 1.35)	7.2^{-15}
Large HDL particles	0.03 (0.01) mmol/L	1.24 (1.18, 1.30)	5.3^{-12}	1.20 (1.14, 1.26)	1.3^{-08}	1.21 (1.15, 1.28)	1.7^{-08}
Medium HDL particles	0.05 (0.02) mmol/L	1.36 (1.30, 1.43)	1.0^{-20}	1.27 (1.21, 1.34)	9.1^{-13}	1.33 (1.26, 1.40)	3.0^{-15}
Small HDL particles	0.05 (0.02) mmol/L	1.52 (1.44, 1.59)	1.9^{-29}	1.35 (1.28, 1.42)	3.1^{-15}	1.48 (1.40, 1.56)	1.9^{-22}
**Phospholipid concentrations**							
Chylomicrons and extremely large VLDL particles	0.04 (0.03) mmol/L	1.53 (1.41, 1.64)	1.7^{-12}	1.33 (1.22, 1.44)	1.0^{-06}	1.49 (1.37, 1.62)	6.8^{-10}
Very large VLDL particles	0.04 (0.03) mmol/L	1.54 (1.43, 1.66)	5.4^{-13}	1.30 (1.19, 1.41)	1.4^{-05}	1.50 (1.37, 1.62)	6.4^{-10}
Large VLDL particles	0.07 (0.04) mmol/L	1.56 (1.44, 1.68)	1.9^{-12}	1.30 (1.18, 1.41)	4.2^{-05}	1.50 (1.37, 1.63)	2.4^{-09}
Medium VLDL particles	0.13 (0.05) mmol/L	1.13 (1.07, 1.20)	4.5^{-04}	1.03 (0.97, 1.10)	4.0^{-01}	1.13 (1.06, 1.21)	1.6^{-03}
Small VLDL particles	0.10 (0.03) mmol/L	1.17 (1.10, 1.24)	9.1^{-06}	1.06 (0.99, 1.12)	1.6^{-01}	1.17 (1.10, 1.24)	6.0^{-05}

Table S1. Distribution of metabolic biomarkers and their associations with incident type 2 diabetes among 50,519 participants in the association analyses population

Metabolic biomarker	Mean (SD)	Hazard ratio (95% CI) per 1-SD higher level on the natural log scale					
		Main model*		Main model + HbA1c		Main model excluding first 3 years of follow-up	
		HR (95% CI) †	p-value §	HR (95% CI) †	p-value §	HR (95% CI) †	p-value §
Very small VLDL particles	0.11 (0.03) mmol/L	1.25 (1.18, 1.31)	1.6^{-11}	1.12 (1.06, 1.18)	7.7^{-04}	1.24 (1.17, 1.31)	1.9^{-09}
IDL particles	0.30 (0.06) mmol/L	0.99 (0.93, 1.06)	8.8^{-01}	0.94 (0.88, 1.00)	7.8^{-02}	1.00 (0.94, 1.07)	9.6^{-01}
Large LDL particles	0.37 (0.08) mmol/L	0.97 (0.91, 1.03)	3.3^{-01}	0.92 (0.86, 0.98)	1.3^{-02}	0.98 (0.91, 1.04)	5.4^{-01}
Medium LDL particles	0.16 (0.04) mmol/L	1.06 (0.99, 1.12)	1.1^{-01}	0.98 (0.91, 1.04)	5.4^{-01}	1.06 (1.00, 1.13)	9.6^{-02}
Small LDL particles	0.09 (0.02) mmol/L	1.08 (1.02, 1.13)	2.1^{-02}	0.99 (0.93, 1.05)	8.4^{-01}	1.08 (1.02, 1.14)	2.7^{-02}
Very large HDL particles	0.08 (0.04) mmol/L	0.93 (0.87, 0.98)	1.4^{-02}	0.94 (0.88, 1.00)	7.0^{-02}	0.93 (0.87, 0.99)	2.0^{-02}
Large HDL particles	0.33 (0.15) mmol/L	0.90 (0.85, 0.96)	6.0^{-04}	0.94 (0.87, 1.01)	1.0^{-01}	0.90 (0.84, 0.95)	5.3^{-04}
Medium HDL particles	0.48 (0.10) mmol/L	1.02 (0.96, 1.08)	5.7^{-01}	1.04 (0.98, 1.11)	2.4^{-01}	1.00 (0.93, 1.07)	9.4^{-01}
Small HDL particles	0.65 (0.09) mmol/L	1.11 (1.05, 1.17)	1.1^{-03}	1.09 (1.03, 1.16)	9.4^{-03}	1.10 (1.03, 1.17)	9.5^{-03}
**Total lipid concentrations**							
Chylomicrons and extremely large VLDL particles	0.23 (0.20) mmol/L	1.44 (1.35, 1.52)	8.4^{-17}	1.28 (1.20, 1.36)	1.7^{-08}	1.43 (1.34, 1.52)	1.0^{-13}
Very large VLDL particles	0.20 (0.13) mmol/L	1.47 (1.39, 1.56)	5.8^{-18}	1.28 (1.20, 1.37)	5.1^{-08}	1.45 (1.35, 1.54)	4.0^{-14}
Large VLDL particles	0.33 (0.17) mmol/L	1.37 (1.30, 1.45)	3.2^{-15}	1.22 (1.14, 1.29)	2.1^{-06}	1.36 (1.27, 1.44)	4.3^{-12}
Medium VLDL particles	0.59 (0.20) mmol/L	1.19 (1.13, 1.26)	7.9^{-07}	1.08 (1.01, 1.15)	4.5^{-02}	1.19 (1.11, 1.26)	1.2^{-05}
Small VLDL particles	0.41 (0.13) mmol/L	1.29 (1.22, 1.36)	1.1^{-12}	1.15 (1.08, 1.22)	2.1^{-04}	1.28 (1.20, 1.35)	3.1^{-10}
Very small VLDL particles	0.37 (0.08) mmol/L	1.22 (1.16, 1.28)	1.3^{-09}	1.10 (1.04, 1.17)	4.5^{-03}	1.21 (1.15, 1.28)	7.2^{-08}
IDL particles	1.28 (0.26) mmol/L	0.98 (0.92, 1.04)	6.0^{-01}	0.93 (0.87, 0.99)	4.4^{-02}	0.99 (0.92, 1.05)	7.6^{-01}
Large LDL particles	1.62 (0.35) mmol/L	1.00 (0.94, 1.06)	8.8^{-01}	0.94 (0.88, 1.00)	8.1^{-02}	1.00 (0.94, 1.07)	9.4^{-01}
Medium LDL particles	0.63 (0.16) mmol/L	1.07 (1.01, 1.13)	4.0^{-02}	0.99 (0.93, 1.05)	7.9^{-01}	1.08 (1.01, 1.15)	4.1^{-02}
Small LDL particles	0.29 (0.06) mmol/L	1.08 (1.02, 1.14)	1.5^{-02}	1.00 (0.94, 1.06)	9.2^{-01}	1.09 (1.02, 1.16)	1.8^{-02}
Very large HDL particles	0.18 (0.08) mmol/L	0.87 (0.80, 0.95)	4.5^{-04}	0.89 (0.82, 0.97)	7.6^{-03}	0.87 (0.79, 0.95)	1.1^{-03}
Large HDL particles	0.67 (0.32) mmol/L	0.83 (0.76, 0.90)	1.1^{-06}	0.89 (0.81, 0.96)	4.3^{-03}	0.82 (0.74, 0.90)	2.0^{-06}
Medium HDL particles	1.03 (0.22) mmol/L	0.97 (0.90, 1.03)	3.8^{-01}	1.01 (0.94, 1.07)	9.0^{-01}	0.95 (0.88, 1.02)	2.3^{-01}
Small HDL particles	1.14 (0.15) mmol/L	1.09 (1.03, 1.15)	8.9^{-03}	1.07 (1.01, 1.13)	6.5^{-02}	1.08 (1.01, 1.15)	3.4^{-02}
**Lipoprotein particle sizes**							
VLDL particles	38.6 (1.2) nm	1.30 (1.23, 1.37)	5.4^{-13}	1.21 (1.14, 1.28)	5.4^{-07}	1.29 (1.21, 1.36)	2.5^{-10}
LDL particles	23.9 (0.1) nm	0.84 (0.78, 0.89)	1.2^{-10}	0.87 (0.82, 0.93)	1.8^{-06}	0.84 (0.79, 0.90)	2.3^{-08}
HDL particles	9.66 (0.21) nm	0.83 (0.74, 0.92)	3.8^{-05}	0.91 (0.82, 1.00)	4.8^{-02}	0.82 (0.72, 0.91)	4.7^{-05}

Table S1. Distribution of metabolic biomarkers and their associations with incident type 2 diabetes among 50,519 participants in the association analyses population

Metabolic biomarker	Mean (SD)	Hazard ratio (95% CI) per 1-SD higher level on the natural log scale					
		Main model*		Main model + HbA1c		Main model excluding first 3 years of follow-up	
		HR (95% CI) †	p-value §	HR (95% CI) †	p-value §	HR (95% CI) †	p-value §
**Apolipoproteins**							
Apolipoprotein A1	1.45 (0.24) g/L	0.94 (0.87, 1.01)	8.0^{-02}	0.98 (0.91, 1.05)	5.9^{-01}	0.92 (0.85, 1.00)	5.3^{-02}
Apolipoprotein B	0.87 (0.19) g/L	1.06 (1.00, 1.12)	7.5^{-02}	0.98 (0.92, 1.04)	5.9^{-01}	1.07 (1.00, 1.13)	7.7^{-02}
Apolipoprotein B to Apolipoprotein A1 ratio	0.62 (0.16)	1.09 (1.03, 1.16)	1.3^{-02}	0.99 (0.93, 1.06)	8.7^{-01}	1.11 (1.03, 1.18)	9.7^{-03}
**Fatty acids**							
Polyunsaturated	5.05 (0.77) mmol/L	1.04 (0.98, 1.10)	2.3^{-01}	1.00 (0.94, 1.06)	9.2^{-01}	1.04 (0.98, 1.11)	2.7^{-01}
Monounsaturated	2.81 (0.79) mmol/L	1.33 (1.27, 1.39)	3.4^{-20}	1.21 (1.15, 1.27)	2.6^{-09}	1.32 (1.26, 1.39)	3.3^{-16}
Saturated	4.07 (0.91) mmol/L	1.33 (1.28, 1.39)	6.8^{-22}	1.21 (1.15, 1.26)	7.6^{-10}	1.31 (1.25, 1.38)	5.0^{-17}
Docosahexaenoic acid	0.24 (0.08) mmol/L	0.91 (0.85, 0.97)	2.5^{-03}	0.93 (0.87, 0.99)	4.2^{-02}	0.90 (0.83, 0.97)	3.4^{-03}
Linoleic acid	3.51 (0.64) mmol/L	1.02 (0.96, 1.08)	5.2^{-01}	0.97 (0.91, 1.03)	3.8^{-01}	1.03 (0.96, 1.09)	4.8^{-01}
Omega-3	0.52 (0.22) mmol/L	1.05 (0.99, 1.12)	1.3^{-01}	1.04 (0.98, 1.10)	3.1^{-01}	1.04 (0.97, 1.11)	2.7^{-01}
Omega-6	4.53 (0.65) mmol/L	1.03 (0.97, 1.09)	3.1^{-01}	0.99 (0.92, 1.05)	6.9^{-01}	1.04 (0.97, 1.10)	2.9^{-01}
Total	11.9 (2.3) mmol/L	1.26 (1.20, 1.32)	6.8^{-14}	1.15 (1.10, 1.21)	5.4^{-06}	1.25 (1.18, 1.31)	4.6^{-11}
Polyunsaturated to total fatty acids ratio	42.8 (3.6) %	0.69 (0.64, 0.74)	2.9^{-44}	0.76 (0.71, 0.81)	1.0^{-22}	0.70 (0.65, 0.76)	5.6^{-34}
Monounsaturated to total fatty acids ratio	23.2 (2.5) %	1.42 (1.36, 1.49)	1.9^{-24}	1.30 (1.23, 1.37)	2.1^{-13}	1.41 (1.34, 1.49)	4.2^{-20}
Saturated to total fatty acids ratio	34.0 (1.9) %	1.40 (1.35, 1.46)	5.9^{-33}	1.28 (1.23, 1.34)	5.8^{-18}	1.37 (1.32, 1.43)	9.0^{-25}
Docosahexaenoic to total fatty acids ratio	2.00 (0.66) %	0.80 (0.74, 0.85)	3.4^{-14}	0.86 (0.80, 0.92)	1.6^{-06}	0.80 (0.74, 0.86)	3.7^{-12}
Linoleic to total fatty acids ratio	29.7 (3.1) %	0.73 (0.68, 0.78)	8.9^{-31}	0.77 (0.72, 0.82)	2.2^{-20}	0.74 (0.69, 0.80)	4.1^{-23}
Omega-3 to total fatty acids ratio	4.33 (1.53) %	0.92 (0.85, 0.98)	1.4^{-02}	0.95 (0.88, 1.02)	2.0^{-01}	0.91 (0.84, 0.98)	1.6^{-02}
Omega-6 to total fatty acids ratio	38.4 (3.4) %	0.72 (0.67, 0.77)	2.3^{-38}	0.78 (0.73, 0.83)	1.3^{-20}	0.73 (0.68, 0.78)	8.9^{-29}
**Other lipids**							
Total cholines	2.57 (0.39) mmol/L	1.08 (1.02, 1.14)	2.6^{-02}	1.04 (0.98, 1.11)	2.4^{-01}	1.07 (1.00, 1.14)	6.1^{-02}
Phosphatidylcholines	2.11 (0.36) mmol/L	1.11 (1.05, 1.17)	2.2^{-03}	1.08 (1.02, 1.15)	2.9^{-02}	1.09 (1.03, 1.16)	1.7^{-02}
Sphingomyelins	0.45 (0.07) mmol/L	0.95 (0.89, 1.01)	1.3^{-01}	0.91 (0.85, 0.98)	8.6^{-03}	0.96 (0.89, 1.03)	3.2^{-01}
Phosphoglycerides	2.28 (0.38) mmol/L	1.13 (1.07, 1.19)	3.0^{-04}	1.09 (1.02, 1.15)	1.9^{-02}	1.12 (1.05, 1.19)	2.5^{-03}
**Glycolysis-related metabolites**							
Lactate	3.77 (1.06) mmol/L	1.16 (1.09, 1.22)	1.4^{-05}	1.10 (1.04, 1.17)	4.1^{-03}	1.13 (1.06, 1.20)	8.1^{-04}
Citrate	0.06 (0.01) mmol/L	1.08 (1.02, 1.15)	1.6^{-02}	1.05 (0.99, 1.11)	1.7^{-01}	1.08 (1.01, 1.15)	3.3^{-02}

Table S1. Distribution of metabolic biomarkers and their associations with incident type 2 diabetes among 50,519 participants in the association analyses population

Metabolic biomarker	Mean (SD)	Hazard ratio (95% CI) per 1-SD higher level on the natural log scale					
		Main model*		Main model + HbA1c		Main model excluding first 3 years of follow-up	
		HR (95% CI) †	p-value §	HR (95% CI) †	p-value §	HR (95% CI) †	p-value §
Glucose	3.41 (0.71) mmol/L	1.65 (1.59, 1.71)	3.2^{-59}	1.28 (1.23, 1.34)	1.0^{-15}	1.57 (1.51, 1.64)	1.6^{-39}
**Amino acids**							
Alanine	0.29 (0.07) mmol/L	1.28 (1.21, 1.35)	3.8^{-11}	1.15 (1.07, 1.22)	5.7^{-04}	1.24 (1.16, 1.32)	2.2^{-07}
Glutamine	0.53 (0.08) mmol/L	0.89 (0.83, 0.94)	9.5^{-05}	0.92 (0.86, 0.98)	7.7^{-03}	0.90 (0.84, 0.96)	2.3^{-03}
Histidine	0.06 (0.01) mmol/L	0.97 (0.91, 1.03)	3.8^{-01}	0.98 (0.92, 1.04)	6.1^{-01}	0.97 (0.91, 1.04)	4.3^{-01}
Glycine	0.17 (0.06) mmol/L	0.82 (0.77, 0.87)	2.8^{-15}	0.85 (0.79, 0.90)	8.1^{-09}	0.82 (0.77, 0.88)	3.7^{-12}
Isoleucine	0.05 (0.02) mmol/L	1.16 (1.09, 1.22)	6.3^{-06}	1.11 (1.04, 1.17)	2.9^{-03}	1.16 (1.10, 1.23)	1.3^{-05}
Leucine	0.10 (0.03) mmol/L	1.15 (1.09, 1.21)	2.0^{-05}	1.12 (1.06, 1.18)	1.1^{-03}	1.15 (1.08, 1.22)	8.1^{-05}
Valine	0.20 (0.04) mmol/L	1.29 (1.23, 1.35)	9.0^{-15}	1.19 (1.13, 1.26)	3.0^{-07}	1.29 (1.23, 1.36)	6.3^{-13}
Phenylalanine	0.04 (0.01) mmol/L	1.12 (1.06, 1.18)	3.7^{-04}	1.06 (1.00, 1.12)	6.9^{-02}	1.13 (1.06, 1.19)	5.3^{-04}
Tyrosine	0.06 (0.01) mmol/L	1.34 (1.28, 1.40)	3.4^{-20}	1.21 (1.15, 1.28)	7.0^{-09}	1.32 (1.26, 1.39)	1.9^{-15}
**Ketone bodies**							
Acetate mmol/l	0.02 (0.01) mmol/L	0.96 (0.91, 1.02)	1.8^{-01}	1.02 (0.96, 1.07)	6.3^{-01}	0.98 (0.92, 1.04)	4.7^{-01}
Acetoacetate mmol/l	0.01 (0.01) mmol/L	1.02 (0.96, 1.08)	5.3^{-01}	1.03 (0.97, 1.10)	4.4^{-01}	1.06 (0.99, 1.12)	1.4^{-01}
3-Hydroxybutyrate mmol/l	0.01 (0.01) mmol/L	0.91 (0.84, 0.97)	4.8^{-03}	0.98 (0.92, 1.05)	6.6^{-01}	0.93 (0.86, 1.00)	4.5^{-02}
Acetone	0.08 (0.03) mmol/L	1.05 (0.99, 1.11)	1.5^{-01}	1.01 (0.94, 1.07)	8.5^{-01}	1.04 (0.97, 1.11)	2.9^{-01}
Pyruvate	0.06 (0.06) mmol/L	0.98 (0.91, 1.04)	5.2^{-01}	0.99 (0.92, 1.06)	7.6^{-01}	0.99 (0.92, 1.06)	8.4^{-01}
**Fluid balance**							
Albumin	39.1 (3.2) g/L	1.03 (0.97, 1.09)	3.4^{-01}	1.11 (1.05, 1.17)	1.4^{-03}	1.04 (0.97, 1.10)	3.4^{-01}
Creatinine	0.07 (0.01) mmol/L	1.06 (0.99, 1.13)	1.0^{-01}	1.04 (0.97, 1.11)	3.6^{-01}	1.07 (1.00, 1.15)	9.5^{-02}
**Inflammation**							
Glycoprotein acetyls	0.78 (0.11) mmol/L	1.30 (1.23, 1.36)	3.3^{-14}	1.06 (1.00, 1.13)	1.1^{-01}	1.31 (1.24, 1.38)	3.1^{-13}

* Main model: Stratified by age-at-risk and sex and adjusted for assessment centre, Townsend deprivation index, ethnicity, parental history of diabetes, smoking, alcohol drinking, physical activity, intakes of whole grains, refined grains, fruit, vegetables, cheese, unprocessed red meat, processed meat, non-oily fish, oily fish, type of spread, coffee (regular and decaffeinated), tea, and dietary supplements, body mass index, waist-to-hip ratio, fasting duration and spectrometer
† HR per 1-SD higher metabolic biomarker on the natural log scale
§ Controlled for false discovery rate

Table S2. Diagnosis and medication codes for assessment of type 2 diabetes status in primary and secondary healthcare and death registry records and UK Biobank verbal interview

Code format	Codes
Diagnosis codes	
**ICD10**	E11
**Read v2**	C1001, C1011, C1021, C1031, C1041, C1051, C1061, C1071, C1074, C109., C1090, C1091, C1092, C1093, C1094, C1095, C1096, C1097, C1099, C109A, C109B, C109C, C109D, C109E, C109F, C109G, C109H, C109J, C109K, C10F., C10F0, C10F1, C10F2, C10F3, C10F4, C10F5, C10F6, C10F7, C10F9, C10FA, C10FB, C10FC, C10FD, C10FE, C10FF, C10FG, C10FH, C10FJ, C10FK, C10FL, C10FM, C10FN, C10FP, C10FQ, C10FR, C10P1, C10y1, C10z1, L1806, L180B
**Read CTv3**	66A3., C1001, C1011, C1021, C1031, C1041, C1051, C1061, C1071, C1074, C109., C1090, C1091, C1092, C1093, C1094, C1095, C1096, C1097, C10y1, C10z1, L1806, X40J5, X40J6, Xaagf, XacsX, XacsY, XaELQ, XaEnp, XaEnq, XaF05, XaFmA, XaFn7, XaFn8, aFn8, XaFn9, XaFWI, XaIfG, XaIfI, XaIzQ, XaIzR, XaJQp, XaKyX, XaXZR, XE10F, XE12A, XE12A, XE12A, XE12A, XM19j
Medication codes*	
**Read v2**	f1..., , f11.., f111., f112., f12.., f121., f122., f123., f124., f125., f126., f127., f128., f129., f12A., f12a., f12B., f12C., f12D., f12d., f12E., f12e., f12F., f12g., f12G., f12H., f12I., f12J., f12K., f12L., f12M., f12Q., f12R., f12s., f12S., f12T., f12U., f12V., f12W., f12X., f12y., f12Y., f12z., f12Z., f13.., f131., f132., f133., f134., f135., f136., f137., f138., f139., f13A., f14.., f141., f142., f143., f144., f145., f146., f14w., f14x., f14y., f14z., f15.., f151., f152., f153., f154., f155., f15x., f15y., f15z., f2..., f21.., f211., f212., f22.., f221., f222., f223., f224., f225., f226., f227., f228., f23.., f231., f24.., f241., f242., f25.., f251., f252., f253., f254., f255., f256., f257., f258., f259., f25a., f25A., f25b., f25B., f25c., f25C., f25d., f25D., f25e., f25E., f25f., f25F., f25g., f25G., f25h., f25H., f25i., f25j., f25k., f25l., f25m., f25n., f25o., f25p., f25q., f25r., f25s., f25t., f25u., f25v., f25W., f25w., f25X., f25x., f25Y., f25y., f25z., f26.., f261., f262., f27.., f271., f272., f273., f274., f275., f276., f277., f278., f279., f27A., f27a., f27b., f27B., f27C., f27c., f27D., f27d., f27E., f27e., f27f., f27F., f27g., f27G., f27h., f27H., f27i., f27I., f27J., f27j., f27k., f27K., f27l., f27L., f27m., f27M., f27n., f27N., f27o., f27O., f27P., f27p., f27Q., f27q., f27R., f27r., f27S., f27s., f27T., f27t., f27u., f27v., f27V., f27W., f27w., f27x., f27X., f27Y., f27y., f27z., f27Z., f28.., f281., f282., f283., f284., f285., f286., f287., f288., f289., f28A., f28B., f28C., f28D., f28E., f28F., f29.., f291., f292., f293., f294., f295., f296., f297., f298., f299., f29A., f29B., f29C., f2A.., f2A1., f2A2., f2A3., f2Ax., f2Ay., f2Az., f2B.., f2B1., f2B2., f2B3., f2B4., f2B5., f2B6., f2C.., f2C1., f2C2., f3..., f31.., f311., f31z., f32.., f321., f322., f323., f324., f325., f33.., f331., f332., f333., f334., f335., f336., f337., f338., f339., f33a., f33b., f33c., f33d., f33e., f33f., f33g., f34.., f341., f34z., f35.., f351., f352., f353., f354., f355., f356., f357., f358., f359., f35A., f35B., 35C., f35D., f35w., f35x., f35y., f35z., f36.., f361., f362., f363., f364., f36y., f36z., f37.., f371., f37z., f38.., f381., f38z., f39.., f391., f392., f39y., f39z., f3A.., f3a.., f3A1., f3a1., f3A2., f3a2., f3a3., f3A3., f3a4., f3A4., f3A5., f3A6., f3A7., f3A8., f3A9., f3AA., f3AB., f3AC., f4..., f41.., f411., f412., f413., f414., f415., f416., f417., f418., f419., f41A., f41B., f41C., f41D., f41E., f41F., f41G., f41H., f41I., f41J., f41s., f41t.,

**Read v2 continued…**	f41u., f41v., f41w., f41x., f41y., f41z., ft1.., ft11., ft12., ft13., ft14., ft2.., ft21., ft22., ft23., ft24., ft25., ft26., ft3.., ft31., ft32., ft33., ft34., ft35., ft36., ft37., ft38., ft39., ft4.., ft41., ft42., ft43., ft44., ft45., ft46., ft4u., ft4v., ft4w., ft4x., ft4y., ft4z., ft5.., ft51., ft52., ft53., ft54., ft55., ft56., ft5x., ft5y., ft5z., ft6.., ft61., ft62., ft63., ft6x., ft6y., ft6z., ft7.., ft71., ft7z., ft8.., ft81., ft82., ft83., ft8x., ft8y., ft8z., ft9.., ft91., ft92., ft93., ft94., ft95., ft9x., ft9y., ft9z., fta.., fta1., ftaZ., ftb.., ftb1., ftb2., ftby., ftbz., ftc.., ftc1., ftc2., ftd.., ftd1., ftd2., ftdy., ftdz., fte.., fte1., ftez., ftf.., ftf1., ftf2., ftg.., ftg1., ftg2., ftg3., ftg4., fth.., fth1., fth2., fth3., fth4., fti.., fti1., fti2., fti3., fti4., ftj.., ftj1., ftj2., ftj3., ftj4., ftj5., ftj6. ftk.., ftk1., ftk2., ftk3., ftk4., ftk5., ftk6., ftl.., ftl1., ftl2., ftm.., ftm1., ftm2., ftm3., ftm4., ftn.., ftn1., ftn2., ftn3., ftn4., fto.., fto1., fto2., fto3., fto4., ftp.., ftp1., ftp2., ftp3., ftp4., ftq.., ftq1., ftq2., ftq3., ftq4., ftq5., ftq6., ftq7., ftq8., ftr.., ftr1., ftr2., ftr3., ftr4., ftr5., ftr6., ftr7., ftr8., fts.., fts1., fts2., fw..., fw1.., fw11., fw12., fw13., fw14., fw15., fw16., fw2.., fw21., fw22., pm1e
**British National Formulary**	0601011A0, 0601011A0, 0601011A0, 0601011A0, 0601011P0, 0601011Q0, 0601011R0, 060101200, 0601012C0, 0601012D0, 0601012F0, 0601012G0, 0601012L0, 0601012N0, 0601012S0, 0601012U0, 0601012V0, 0601012W0, 0601012X0, 0601012Z0, 0601021A0, 0601021B0, 0601021E0, 0601021H0, 0601021K0, 0601021M0, 0601021P0, 0601021R0, 0601021T0, 0601021V0, 0601021X0, 0601022B0, 0601022P0, 0601023A0, 0601023AA, 0601023AB, 0601023AC, 0601023AD, 0601023AE, 0601023AF, 0601023AG, 0601023AH, 0601023AI, 0601023AJ, 0601023AK, 0601023AL, 0601023AM, 0601023AN, 0601023AP, 0601023AQ, 0601023AR, 0601023AS, 0601023AU, 0601023AV, 0601023AW, 0601023AX, 0601023B0, 0601023M0, 0601023R0, 0601023S0, 0601023T0, 0601023U0, 0601023V0, 0601023W0, 0601023X0, 0601023Y0,0601023Z0
**UKB verbal interview**	1140857494, 1140857496, 1140857500, 1140857502, 1140857506, 1140857584, 1140857586, 1140857590, 1140868902, 1140868908, 1140874646, 1140874650, 1140874652, 1140874658, 1140874660, 1140874664, 1140874666, 1140874674, 1140874678, 1140874680, 1140874686, 1140874690, 1140874706, 1140874712, 1140874716, 1140874718, 1140874724, 1140874726, 1140874728, 1140874732, 1140874736, 1140874740, 1140874744, 1140874746, 1140882964, 1140883066, 1140884600, 1140910564, 1140910566, 1140910818, 1140921964, 1141152590, 1141153254, 1141153262, 1141156984, 1141157284, 1141168660, 1141168668, 1141169504, 1141171508, 1141171646, 1141171652, 1141173786, 1141173882, 1141177600, 1141177606, 1141189090, 1141189094

* Participants for whom receiving metformin (self-reported use or recorded in primary care data) was the only evidence of a diagnosis of diabetes were not considered to have diabetes given other indications for its use. Participants for whom receiving insulin (self-reported use or a prescription recorded in primary care data) was the only evidence of a diagnosis of diabetes were not considered to have type 2 diabetes.

Table S3. Baseline characteristics of participants with and without NMR-metabolomics profiling

**Baseline characteristics***	**Included in NMR-metabolomics profiling**	
	**Yes**	**No**
**No. of participants**	118036	384454
**Age, sex and socioeconomic factors**		
Mean age (SD), years	56.5 (8.1)	56.5 (8.1)
Women, %	54	54
Townsend Deprivation Index (SD)†	0.0 (1.0)	0.0 (1.0)
**Lifestyle factors**		
Smoking, %		
Never or occasional	57	58
Previous	35	34
Current regular	8	8
Alcohol drinking, %		
Never or occasional	27	27
Previous	4	4
Current regular	69	69
**Anthropometry, mean (SD)**		
BMI, kg/m^2	27.4 (4.8)	27.4 (4.8)
WC, cm	90 (13)	90 (14)
HC, cm	103 (9)	103 (9)
WHR	0.87 (0.09)	0.87 (0.09)
**Parental history of diabetes, %**	20	20
**Mean fasting time (SD), hours**	3.8 (2.4)	3.8 (2.5)

* Standardised to age and sex structure of the study population

† Standardised Townsend Deprivation Index, higher scores represent higher levels of deprivation

BMI=body mass index; HC=hip circumference; WC=waist circumference; WHR=waist-to-hip ratio

Participants with missing data: age n=1; sex n=1; Townsend Deprivation Index n=624; smoking n=1171; alcohol n=1654; BMI n=10136; WC n=2161; HC n=2220; WHR n=2226; parental history of diabetes n=58299; fasting time n=1234

Table S4. Baseline characteristics of 50,519 participants in the association analyses population by incident type 2 diabetes status

Baseline characteristics*	Incident type 2 diabetes		Total
	Yes	No	
**No. of participants**	1211	49308	50519
**Age, sex and socioeconomic factors**			
Mean age (SD), years	57.1 (7.8)	54.8 (8.0)	54.9 (8.0)
Women, %	44	56	55
Townsend Deprivation Index (SD) †	0.2 (1.1)	0.0 (1.0)	0.0 (1.0)
White ethnicity, %	91	96	96
**Lifestyle factors**			
Smoking, %			
Never or occasional	53	61	61
Previous	34	32	32
Current regular	13	7	7
Alcohol drinking, %			
Never or occasional	39	23	24
Previous	5	3	3
Current regular	55	74	73
Regular dietary consumption, %			
Fresh fruit ‡	60	65	65
Vegetables ‡	63	65	65
Non-oily fish §	65	66	66
Oily fish §	51	55	55
Unprocessed red meat §	91	90	90
Processed meat §	66	60	60
Cheese ||	54	62	62
Butter	38	39	39
Whole grain foods #	52	64	64
Refined grain foods #	42	33	33
Coffee $			
Caffeinated	61	65	65
Decaffeinated	13	14	14
Tea $	81	86	86
Dietary supplement use, %	46	48	48
Physical activity, MET-h/week	39.3 (46.0)	44.4 (43.8)	44.3 (43.8)
**Anthropometry, mean (SD)**			
BMI, kg/m^2	31.5 (5.2)	26.7 (4.3)	26.8 (4.3)
WC, cm	100 (13)	88 (12)	88 (13)
HC, cm	109 (10)	102 (8)	103 (8)
WHR	0.91 (0.08)	0.86 (0.09)	0.86 (0.09)
**Parental history of diabetes, %**	39	18	18
**Mean fasting time (SD), hours**	4.0 (2.5)	3.7 (2.4)	3.7 (2.4)

* Standardised to age and sex structure of the study population
† Standardised Townsend Deprivation Index, higher scores represent higher levels of deprivation
‡ Consumption of ≥2 servings/day
§ Consumption ≥1 time/week
|| Consumption ≥2 times/week
Consumption ≥1 time/day
$ Consumption ≥1 cup/day
BMI=body mass index; HC=hip circumference; MET-h=metabolic equivalent of task hours; WC=waist circumference; WHR=waist-to-hip ratio

Table S5. Associations of the first 11 metabolic biomarker principal components with risk of incident type 2 diabetes among 50,519 participants in the association analyses population

Principal component	Main model* HR (95% CI)	Main model + sequential adjustment † HR (95% CI)
1	1.25 (1.17, 1.34)	1.25 (1.17, 1.34)
2	0.81 (0.76, 0.87)	0.74 (0.69, 0.80)
3	1.23 (1.17, 1.30)	1.19 (1.13, 1.25)
4	1.13 (1.06, 1.20)	1.08 (1.01, 1.15)
5	1.07 (1.00, 1.14)	1.08 (1.02, 1.15)
6	0.95 (0.89, 1.01)	0.98 (0.92, 1.05)
7	0.72 (0.67, 0.76)	0.75 (0.70, 0.80)
8	0.84 (0.79, 0.89)	0.88 (0.83, 0.94)
9	0.94 (0.89, 1.00)	0.91 (0.85, 0.96)
10	0.88 (0.83, 0.93)	0.94 (0.88, 0.99)
11	0.93 (0.88, 0.99)	0.99 (0.94, 1.05)

* Hazard ratios (95% CI) stratified by age-at-risk and sex and adjusted for assessment centre, Townsend deprivation index, ethnicity, parental history of diabetes, smoking, alcohol drinking, intake of whole grains, refined grains, fruit, vegetables, cheese, unprocessed red meat, processed meat, non-oily fish, oily fish, type of spread, coffee (regular and decaffeinated), tea, and dietary supplements, physical activity, body mass index, waist-to-hip ratio, fasting duration, and spectrometer.

† Hazard ratios (95% CI) stratified by age-at-risk and sex and adjusted for assessment centre, Townsend deprivation index, ethnicity, parental history of diabetes, smoking, alcohol drinking, intake of whole grains, refined grains, fruit, vegetables, cheese, unprocessed red meat, processed meat, non-oily fish, oily fish, type of spread, coffee (regular and decaffeinated), tea, and dietary supplements, physical activity, body mass index, waist-to-hip ratio, fasting duration, spectrometer, and preceding principal components. E.g. for principal component 3 the hazard ratio is adjusted for these same variables plus principal component 1 and principal component 2.

Table S6. Regression models for risk of incident type 2 diabetes among 65,684 participants in the risk prediction population

	Concise model* HR (95% CI)	Concise model* plus metabolic biomarkers † HR (95% CI)	Full model ‡ HR (95% CI)	Full model ‡ plus metabolic biomarkers † HR (95% CI)
Age (years)				
<50	1.00 (reference)	1.00 (reference)	1.00 (reference)	1.00 (reference)
50 to <65	1.45 (1.28, 1.64)	1.47 (1.30, 1.67)	1.40 (1.23, 1.59)	1.40 (1.24, 1.59)
≥65	2.06 (1.77, 2.40)	2.10 (1.80, 2.46)	1.90 (1.62, 2.22)	1.91 (1.63, 2.24)
Sex				
Women	1.00 (reference)	1.00 (reference)	1.00 (reference)	1.00 (reference)
Men	1.56 (1.42, 1.72)	1.06 (0.94, 1.18)	1.62 (1.47, 1.79)	1.27 (1.12, 1.44)
Parental history of diabetes				
No	1.00 (reference)	1.00 (reference)	1.00 (reference)	1.00 (reference)
Yes	1.92 (1.74, 2.13)	1.86 (1.68, 2.06)	1.87 (1.69, 2.06)	1.85 (1.67, 2.05)
Body mass index (kg/m²)				
<25	1.00 (reference)	1.00 (reference)	1.00 (reference)	1.00 (reference)
25 to <30	2.45 (2.07, 2.90)	1.83 (1.54, 2.18)	1.64 (1.38, 1.96)	1.52 (1.27, 1.82)
≥30	6.69 (5.68, 7.88)	3.90 (3.27, 4.65)	2.68 (2.19, 3.29)	2.32 (1.88, 2.86)
HbA1c (%)				
<6.0 (normal)	1.00 (reference)	1.00 (reference)	1.00 (reference)	1.00 (reference)
≥6.0 (pre-diabetes)	10.14 (9.07, 11.35)	8.66 (7.73, 9.70)	8.84 (7.89, 9.89)	8.30 (7.41, 9.30)
Principal components				
1		1.26 (1.18, 1.33)		1.16 (1.08, 1.25)
2		0.72 (0.67, 0.76)		0.82 (0.76, 0.88)
3		1.12 (1.08, 1.17)		1.11 (1.06, 1.17)
4		1.01 (0.96, 1.06)		1.00 (0.95, 1.05)
5		1.08 (1.03, 1.13)		1.07 (1.02, 1.12)
6		1.01 (0.96, 1.05)		1.00 (0.96, 1.05)
7		0.76 (0.72, 0.80)		0.80 (0.75, 0.84)
8		0.92 (0.87, 0.97)		0.93 (0.88, 0.97)
9		0.93 (0.88, 0.97)		0.92 (0.87, 0.96)
10		0.93 (0.88, 0.97)		0.94 (0.89, 0.98)
11		1.01 (0.97, 1.06)		1.02 (0.98, 1.07)
Blood pressure				
SBP ≤130 mmHg and DBP ≤85 mmHg and no antihypertensive medications			1.00 (reference)	1.00 (reference)
SBP >130 mmHg or DBP >85 mmHg or on antihypertensive medication			1.48 (1.30, 1.69)	1.42 (1.24, 1.62)
Waist circumference (cm)				
≤102 in men; ≤88 in women			1.00 (reference)	1.00 (reference)
>102 in men; >88 in women			1.83 (1.60, 2.09)	1.71 (1.50, 1.96)
HDL-cholesterol (mmol/L)				
≥1.0344 in men; ≥1.293 in women			1.00 (reference)	1.00 (reference)
<1.0344 in men; <1.293 in women			1.79 (1.62, 1.98)	1.38 (1.21, 1.58)
Triglycerides (mmol/L)				
<1.6935			1.00 (reference)	1.00 (reference)
≥1.6935			1.56 (1.40, 1.74)	1.12 (0.96, 1.30)

* Concise model: Hazard ratios (95% CI) adjusted for age, sex, parental history of diabetes, BMI and HbA1c
† Metabolic biomarkers comprise the first 11 metabolic biomarker principal components
‡ Full model: Hazard ratios (95% CI) adjusted for age, sex, parental history of diabetes, BMI, HbA1c, waist circumference, blood pressure, triglycerides, HDL-cholesterol
DBP= diastolic blood pressure; SBP= systolic blood pressure

Table S7. Performance of risk prediction models for incident type 2 diabetes among 13,695 participants taking lipid-lowering medication at recruitment

Performance metric	Concise model*	Concise model* plus metabolic biomarkers †	Full model ‡	Full model ‡ plus metabolic biomarkers †
**C-statistic (CI) §**	0.761 (0.747, 0.777)	0.789 (0.779, 0.806)	0.782 (0.768, 0.798)	0.793 (0.783, 0.810)
**Metrics of relative performance**				
χ^2 || #	174 (p<0.0001)		88 (p<0.0001)	
%increase χ^2	15		7	
Absolute IDI # $	1.3 (0.6, 2.0)		0.7 (0.3, 1.1)	
Relative IDI (%) (CI) # $	9.1 (4.7, 14.2)		4.4 (1.8, 8.0)	
Continuous NRI (CI) # **				
Events	0.13 (0.10, 0.21)		0.07 (0.03, 0.15)	
Non-events	0.24 (0.20, 0.29)		0.13 (0.08, 0.17)	
Overall	0.38 (0.33, 0.48)		0.21 (0.11, 0.29)	

* Concise model: age, sex, parental history of diabetes, body mass index, HbA1c
† Metabolic biomarkers comprise the first 11 metabolic biomarker principal components
‡ Full model: Concise model plus waist circumference, blood pressure, triglycerides, HDL-cholesterol
§ The **c-statistic** measures the ability of a model to rank participants from low to high risk. Given two randomly selected individuals, one who develops T2D and one who does not, the c-statistic is the probability that the model will give a higher predicted risk for the individual who develops T2D. An uninformative model will have a c-statistic of 0.5 and a model that discriminates perfectly will have a c-statistic of 1.0.
|| 11 DF
Bias-corrected estimates and confidence intervals were derived using 200 bootstrap samples
$ The **IDI** quantifies the difference between two models in their ability to predict risk. It is calculated as the difference between the two models in the mean predicted T2D risk among those who did develop T2D minus the mean predicted risk of T2D in those who did not develop T2D (i.e., it is the difference between two differences). When metabolic biomarkers were added to the Concise model, the separation in mean predicted T2D risk between those who did develop T2D, compared with those who did not develop T2D, increased in relative terms by 9.1%. Positive IDI values indicate improved T2D risk classification following addition of metabolic biomarkers to the risk prediction model.
** The continuous **NRI** quantifies the appropriateness of the change in predicted probabilities of T2D between two models. The ‘Events’ NRI is calculated among those who developed T2D, and the ‘Non-events’ NRI is calculated among those who did not develop T2D. Both statistics are calculated as the probability of an ‘appropriate’ change in predicted risk (after addition of metabolic biomarkers to the model) minus the probability of an ‘inappropriate’ change in predicted risk. For those who developed T2D, an appropriate change would be a higher predicted T2D risk after addition of metabolic biomarkers to the model. An inappropriate change would be a lower predicted T2D risk after addition of metabolic biomarkers to the model. When metabolic biomarkers were added to the Concise model, among those who developed T2D, 13% more were assigned a higher predicted T2D risk than were assigned a lower predicted risk. The overall NRI is the sum of the ‘Events’ and ‘Non-events’ NRI statistics. Positive NRI values indicate that addition of metabolic biomarkers results in a superior model.
DF= degrees of freedom; IDI= integrated discrimination improvement; NRI= net reclassification improvement; T2D= type 2 diabetes

Table S8. Performance of risk prediction models including waist-to-hip ratio for incident type 2 diabetes among 65,684 participants in the risk prediction population

Performance metric	Concise model*	Concise model* plus metabolic biomarkers †	Full model ‡	Full model ‡ plus metabolic biomarkers †
**C-statistic (CI) §**	0.802 (0.792, 0.812)	0.830 (0.822, 0.839)	0.829 (0.820, 0.839)	0.837 (0.828, 0.846)
**Metrics of relative performance**				
χ^2 || #	453 (p<0.0001)		178 (p<0.0001)	
%increase χ^2	17		6	
Absolute IDI # $	1.5 (1.0, 1.9)		0.7 (0.4, 1.1)	
Relative IDI (%) (CI) # $	15.1 (10.9, 21.2)		6.5 (4.2, 10.2)	
Continuous NRI (CI) # **				
Events	0.15 (0.10, 0.20)		0.09 (0.03, 0.13)	
Non-events	0.28 (0.26, 0.31)		0.12 (0.09, 0.14)	
Overall	0.44 (0.38, 0.49)		0.20 (0.13, 0.26)	

* Concise model: age, sex, parental history of diabetes, body mass index, HbA1c
† Metabolic biomarkers comprise the first 11 metabolic biomarker principal components
‡ Full model: Concise model plus waist-to-hip ratio (men: <0.90 vs ≥0.9; women <0.85 vs ≥0.85), blood pressure, triglycerides, HDL-cholesterol
§ The **c-statistic** measures the ability of a model to rank participants from low to high risk. Given two randomly selected individuals, one who develops T2D and one who does not, the c-statistic is the probability that the model will give a higher predicted risk for the individual who develops T2D. An uninformative model will have a c-statistic of 0.5 and a model that discriminates perfectly will have a c-statistic of 1.0.
|| 11 DF
Bias-corrected estimates and confidence intervals were derived using 200 bootstrap samples
$ The **IDI** quantifies the difference between two models in their ability to predict risk. It is calculated as the difference between the two models in the mean predicted T2D risk among those who did develop T2D minus the mean predicted risk of T2D in those who did not develop T2D (i.e., it is the difference between two differences). When metabolic biomarkers were added to the Concise model, the separation in mean predicted T2D risk between those who did develop T2D, compared with those who did not develop T2D, increased in relative terms by 15.1%. Positive IDI values indicate improved T2D risk classification following addition of metabolic biomarkers to the risk prediction model.
** The continuous **NRI** quantifies the appropriateness of the change in predicted probabilities of T2D between two models. The ‘Events’ NRI is calculated among those who developed T2D, and the ‘Non-events’ NRI is calculated among those who did not develop T2D. Both statistics are calculated as the probability of an ‘appropriate’ change in predicted risk (after addition of metabolic biomarkers to the model) minus the probability of an ‘inappropriate’ change in predicted risk. For those who developed T2D, an appropriate change would be a higher predicted T2D risk after addition of metabolic biomarkers to the model. An inappropriate change would be a lower predicted T2D risk after addition of metabolic biomarkers to the model. When metabolic biomarkers were added to the Concise model, among those who developed T2D, 15% more were assigned a higher predicted T2D risk than were assigned a lower predicted risk. The overall NRI is the sum of the ‘Events’ and ‘Non-events’ NRI statistics. Positive NRI values indicate that addition of metabolic biomarkers results in a superior model.
DF= degrees of freedom; IDI= integrated discrimination improvement; NRI= net reclassification improvement; T2D= type 2 diabetes

Table S9. Performance of risk prediction models for incident type 2 diabetes incorporating co-variates, where relevant, as continuous variables among 65,684 participants in the risk prediction population

Performance metric	Concise model*	Concise model* plus metabolic biomarkers †	Full model ‡	Full model ‡ plus metabolic biomarkers †
**C-statistic (CI) §**	0.862 (0.854, 0.870)	0.873 (0.866, 0.883)	0.875 (0.867, 0.883)	0.878 (0.871, 0.886)
**Metrics of relative performance**				
χ^2 || #	293 (p<0.0001)		107 (p<0.0001)	
%increase χ^2	7		2	
Absolute IDI # $	1.3 (0.9, 1.9)		0.5 (0.1, 0.8)	
Relative IDI (%) (CI) # $	8.5 (5.5, 12.5)		3.0 (1.6, 5.1)	
Continuous NRI (CI) # **				
Events	0.10 (0.06, 0.18)		0.08 (0.03, 0.13)	
Non-events	0.24 (0.15, 0.29)		0.03 (-0.06, 0.07)	
Overall	0.34 (0.28, 0.41)		0.11 (0.03, 0.17)	

* Concise model: age, sex, parental history of diabetes, body mass index (continuous), HbA1c (continuous)
† Metabolic biomarkers comprise the first 11 metabolic biomarker principal components
‡ Full model: Concise model plus waist circumference, blood pressure, triglycerides, HDL-cholesterol (all as continuous variables)
§ The **c-statistic** measures the ability of a model to rank participants from low to high risk. Given two randomly selected individuals, one who develops T2D and one who does not, the c-statistic is the probability that the model will give a higher predicted risk for the individual who develops T2D. An uninformative model will have a c-statistic of 0.5 and a model that discriminates perfectly will have a c-statistic of 1.0.
|| 11 DF
Bias-corrected estimates and confidence intervals were derived using 200 bootstrap samples
$ The **IDI** quantifies the difference between two models in their ability to predict risk. It is calculated as the difference between the two models in the mean predicted T2D risk among those who did develop T2D minus the mean predicted risk of T2D in those who did not develop T2D (i.e., it is the difference between two differences). When metabolic biomarkers were added to the Concise model, the separation in mean predicted T2D risk between those who did develop T2D, compared with those who did not develop T2D, increased in relative terms by 8.5%. Positive IDI values indicate improved T2D risk classification following addition of metabolic biomarkers to the risk prediction model.
** The continuous **NRI** quantifies the appropriateness of the change in predicted probabilities of T2D between two models. The 'Events' NRI is calculated among those who developed T2D, and the 'Non-events' NRI is calculated among those who did not develop T2D. Both statistics are calculated as the probability of an 'appropriate' change in predicted risk (after addition of metabolic biomarkers to the model) minus the probability of an 'inappropriate' change in predicted risk. For those who developed T2D, an appropriate change would be a higher predicted T2D risk after addition of metabolic biomarkers to the model. An inappropriate change would be a lower predicted T2D risk after addition of metabolic biomarkers to the model. When metabolic biomarkers were added to the Concise model, among those who developed T2D, 10% more were assigned a higher predicted T2D risk than were assigned a lower predicted risk. The overall NRI is the sum of the 'Events' and 'Non-events' NRI statistics. Positive NRI values indicate that addition of metabolic biomarkers results in a superior model.
DF= degrees of freedom; IDI= integrated discrimination improvement; NRI= net reclassification improvement; T2D= type 2 diabetes

Figure S1. Participant exclusions to derive risk prediction and association analyses populations

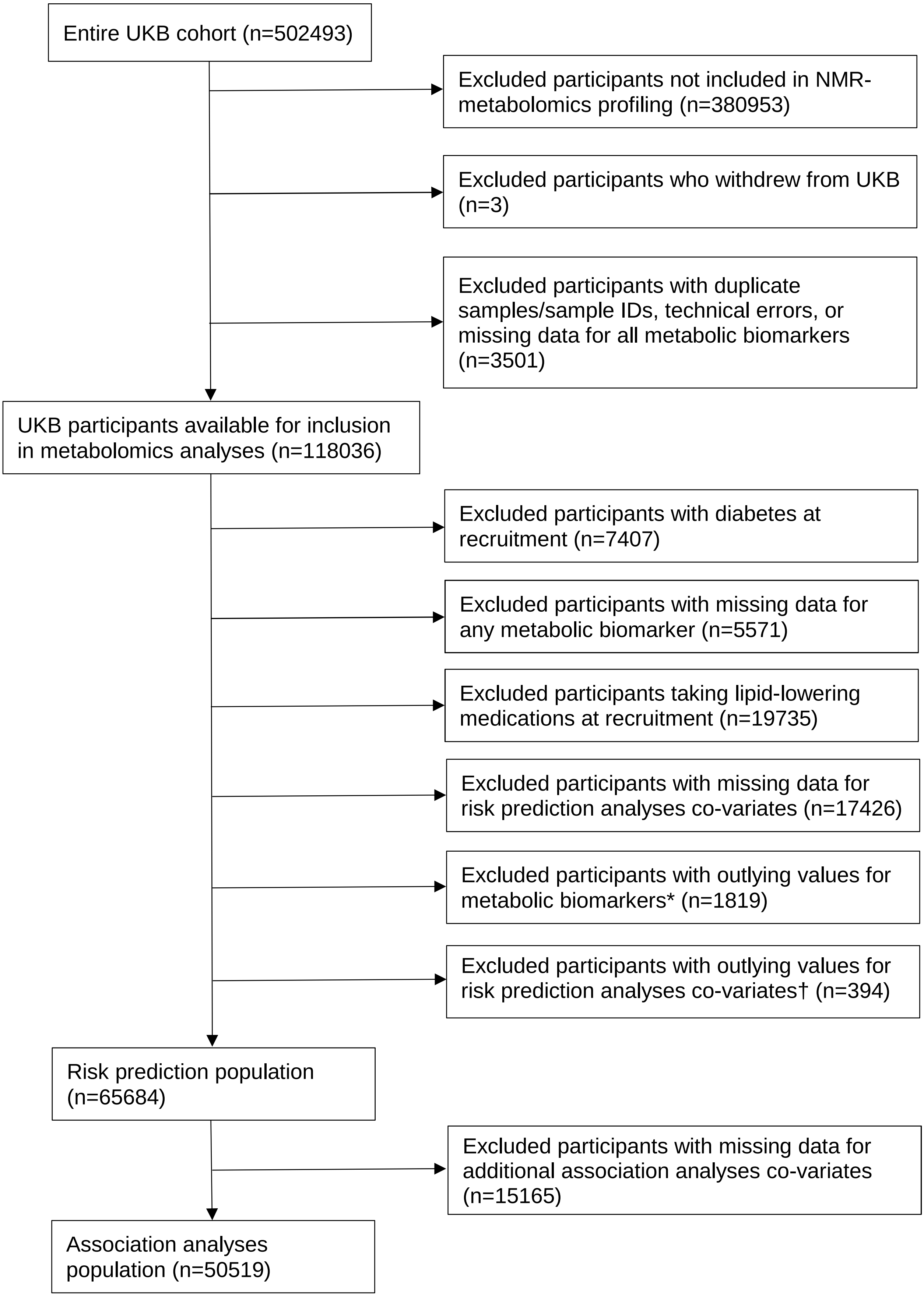

* Values >4 standard deviations from the mean and within the top or bottom 0.003% of the original or log-transformed metabolic biomarker distribution

† Weight <40 or ≥150 kg; height <140 or ≥200 cm; BMI <15 or ≥50 kg/m2; WC <55 or ≥145 cm; HC <75 or ≥150 cm; WHR <0.60 or ≥1.15

BMI=body mass index; DBP=diastolic blood pressure; HC=hip circumference; HDL-C=HDL-cholesterol from routine clinical chemistry measures; SBP=systolic blood pressure; TG=triglycerides from routine clinical chemistry measures; UKB=UK Biobank; WC=waist circumference

Figure S2. Cross−correlations of metabolic biomarkers

a) Lipoproteins, fatty acids and other lipids, and small molecules **b) Lipoproteins, lipids by lipoprotein subclasses, particle sizes, and apolipoproteins**

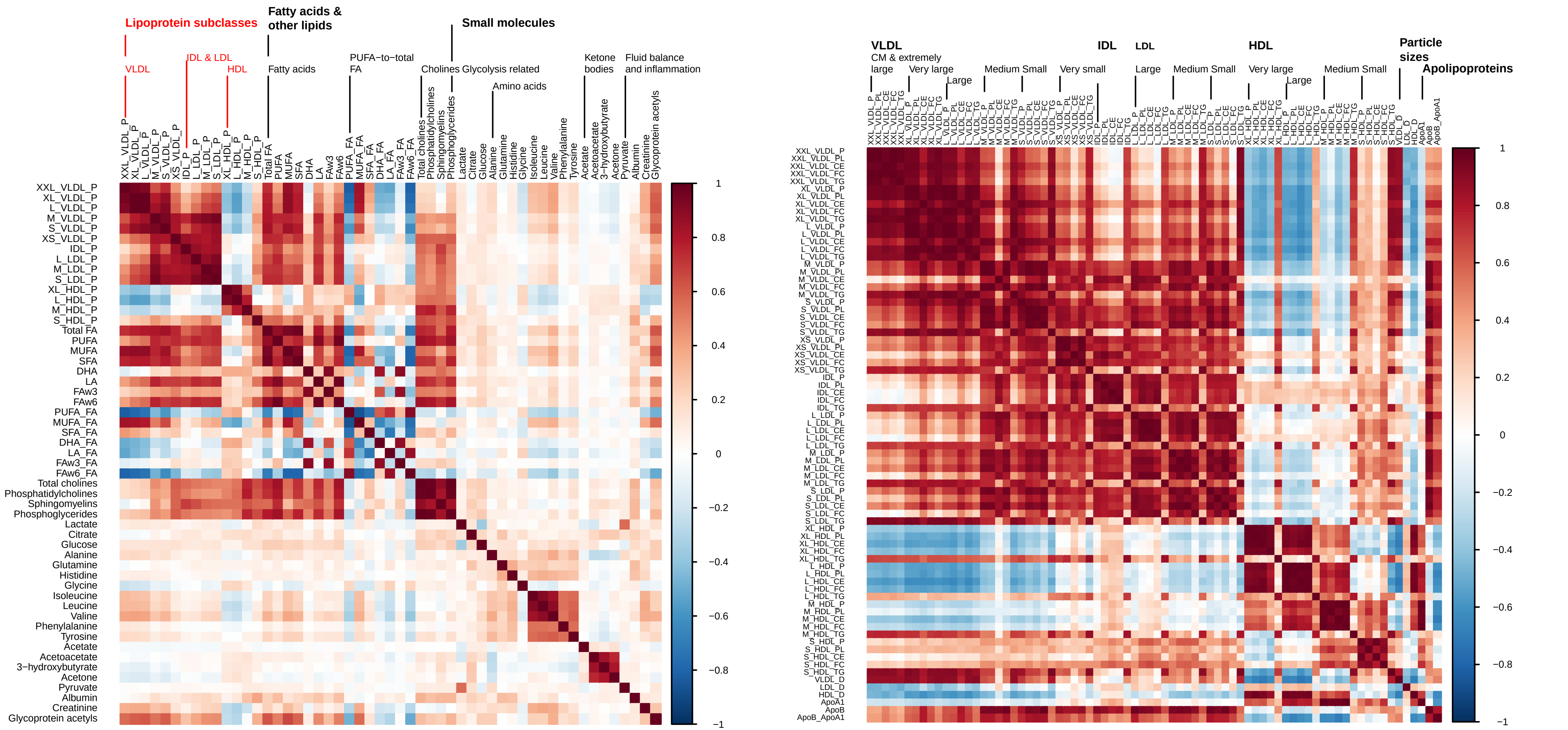

Apo−A1=apolipoprotein A1; Apo−B=apolipoprotein B; DHA=docosahexaenoic acid; FA=fatty acids; FAw3=omega−3 fatty acids; FAw6=omega−6 fatty acids; HDL=high density lipoproteins; HDL−D=high density lipoprotein particle diameter; IDL=intermediate density lipoproteins; L=large; LA=linoleic acid; LDL=low density lipoproteins; LDL−D=low density lipoprotein particle diameter; LP=lipoprotein; M=medium; MUFA=monounsaturated fatty acids; PUFA=polyunsaturated fatty acids; S=small; SFA=saturated fatty acids; T2D=type 2 diabetes; VLDL=very low density lipoproteins; VLDL−D=very low density lipoprotein particle diameter; XL=very large; XS=very small; XXL=extremely large

Figure S3. Associations of metabolic biomarkers with risk of incident type 2 diabetes among 50,519 participants in the association analyses population

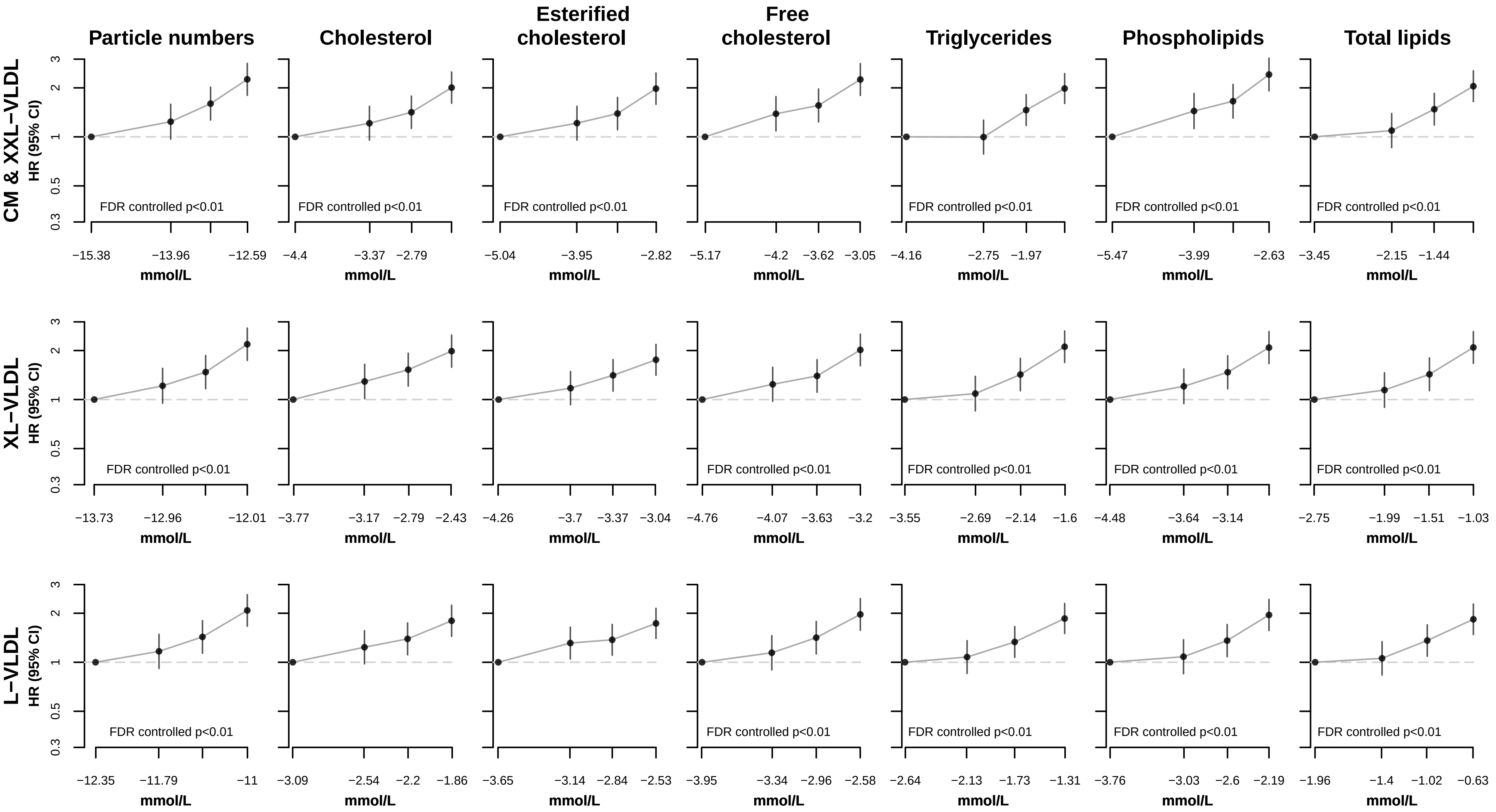

Hazard ratios (HR) stratified by age−at−risk and sex and adjusted for assessment centre, Townsend deprivation index, ethnicity, parental history of diabetes, smoking ,alcohol drinking, intake of whole grains, refined grains, fruit, vegetables, cheese, unprocessed red meat, processed meat, non−oily fish, oily fish, type of spread, coffee (regular and decaffeinated), tea, and dietary supplements, physical activity, body mass index, waist−to−hip ratio, fasting duration, and spectorometer.

Numbers on the x−axis correspond to median values within each quartile for each metabolic biomarker on the natural log scale.

Circles represent the HR and vertical lines indicate the 95% CI.

FDR controlled p values represent the p for trend across quartiles.

CM= chylomicrons; FA= fatty acids; HDL= high density lipoproteins; IDL= intermediate density lipoproteins; L= large; LDL= low density liporpoteins; M= medium; S= small; VLDL= very low density lipoproteins; XL= very large; XS= very small; XXL= extremely large

Figure S3. Associations of metabolic biomarkers with risk of incident type 2 diabetes among 50,519 participants in the association analyses population

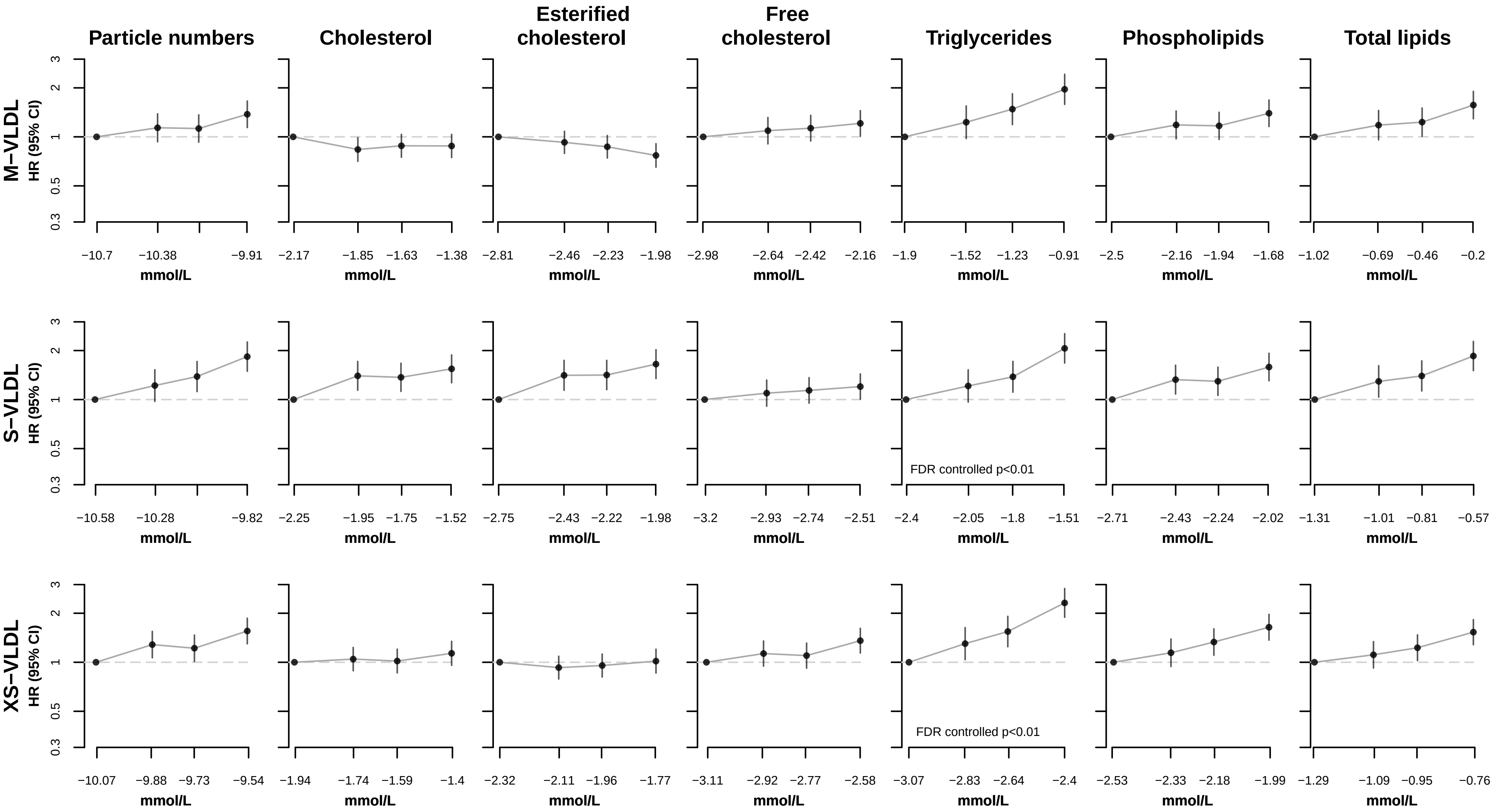

Hazard ratios (HR) stratified by age−at−risk and sex and adjusted for assessment centre, Townsend deprivation index, ethnicity, parental history of diabetes, smoking ,alcohol drinking, intake of whole grains, refined grains, fruit, vegetables, cheese, unprocessed red meat, processed meat, non−oily fish, oily fish, type of spread, coffee (regular and decaffeinated), tea, and dietary supplements, physical activity, body mass index, waist−to−hip ratio, fasting duration, and spectorometer.
Numbers on the x−axis correspond to median values within each quartile for each metabolic biomarker on the natural log scale.
Circles represent the HR and vertical lines indicate the 95% CI.
FDR controlled p values represent the p for trend across quartiles.
CM= chylomicrons; FA= fatty acids; HDL= high density lipoproteins; IDL= intermediate density lipoproteins; L= large; LDL= low density liporpoteins; M= medium; S= small; VLDL= very low density lipoproteins; XL= very large; XS= very small; XXL= extremely large

Figure S3. Associations of metabolic biomarkers with risk of incident type 2 diabetes among 50,519 participants in the association analyses population

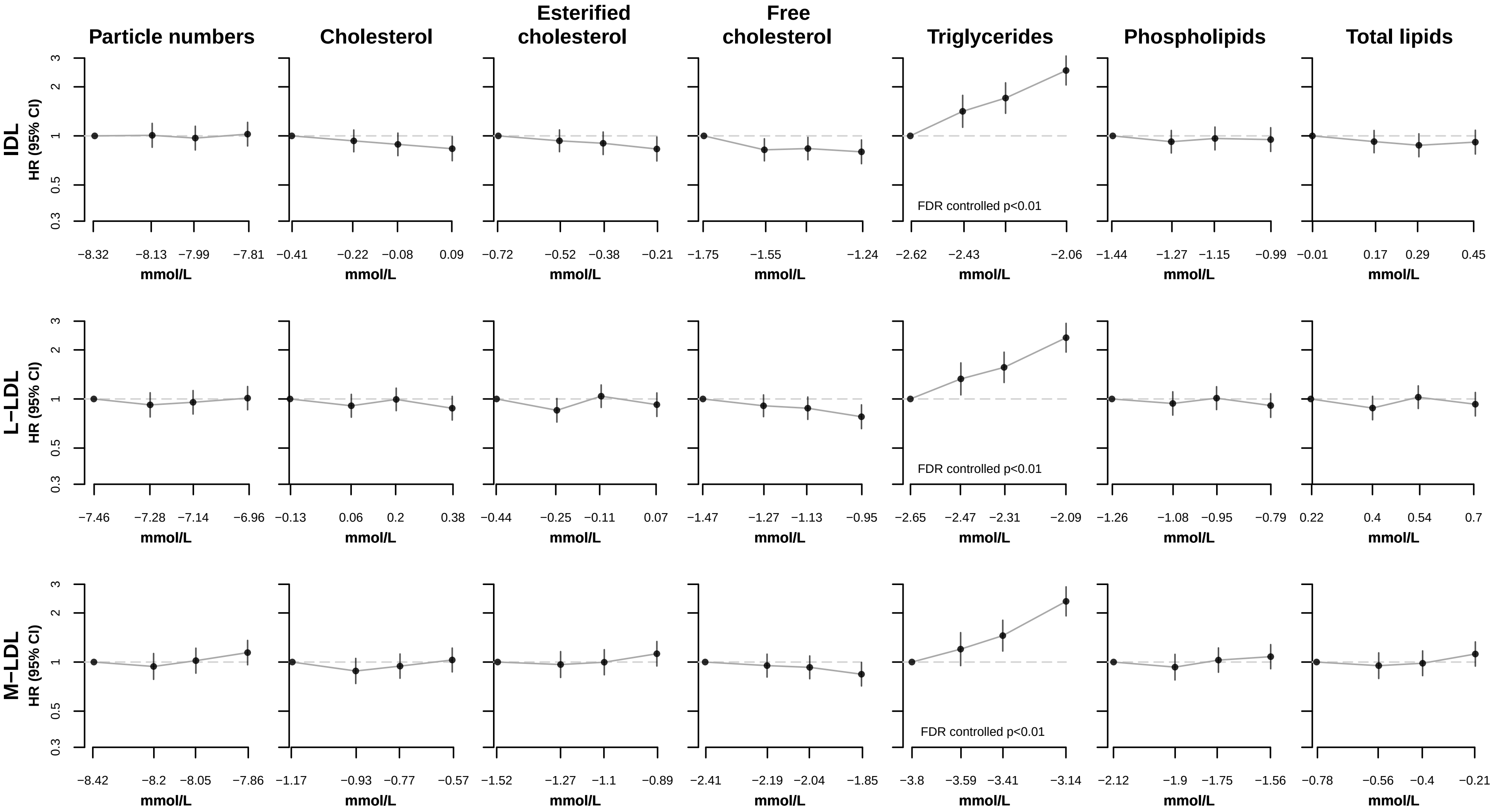

Hazard ratios (HR) stratified by age−at−risk and sex and adjusted for assessment centre, Townsend deprivation index, ethnicity, parental history of diabetes, smoking ,alcohol drinking, intake of whole grains, refined grains, fruit, vegetables, cheese, unprocessed red meat, processed meat, non−oily fish, oily fish, type of spread, coffee (regular and decaffeinated), tea, and dietary supplements, physical activity, body mass index, waist−to−hip ratio, fasting duration, and spectorometer.
Numbers on the x−axis correspond to median values within each quartile for each metabolic biomarker on the natural log scale.
Circles represent the HR and vertical lines indicate the 95% CI.
FDR controlled p values represent the p for trend across quartiles.
CM= chylomicrons; FA= fatty acids; HDL= high density lipoproteins; IDL= intermediate density lipoproteins; L= large; LDL= low density liporpoteins; M= medium; S= small; VLDL= very low density lipoproteins; XL= very large; XS= very small; XXL= extremely large

Figure S3. Associations of metabolic biomarkers with risk of incident type 2 diabetes among 50,519 participants in the association analyses population

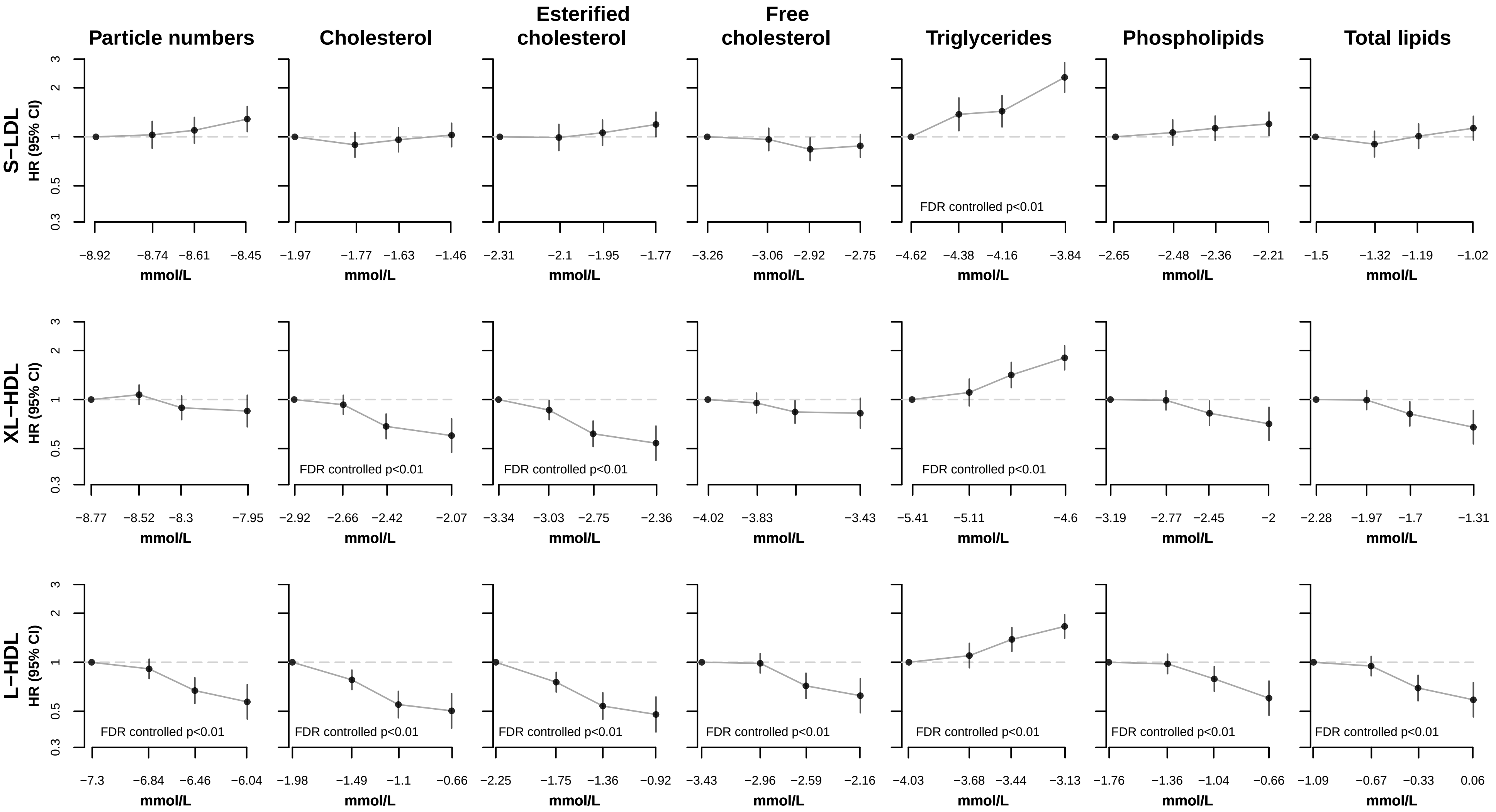

Hazard ratios (HR) stratified by age−at−risk and sex and adjusted for assessment centre, Townsend deprivation index, ethnicity, parental history of diabetes, smoking ,alcohol drinking, intake of whole grains, refined grains, fruit, vegetables, cheese, unprocessed red meat, processed meat, non−oily fish, oily fish, type of spread, coffee (regular and decaffeinated), tea, and dietary supplements, physical activity, body mass index, waist−to−hip ratio, fasting duration, and spectorometer.
Numbers on the x−axis correspond to median values within each quartile for each metabolic biomarker on the natural log scale.
Circles represent the HR and vertical lines indicate the 95% CI.
FDR controlled p values represent the p for trend across quartiles.
CM= chylomicrons; FA= fatty acids; HDL= high density lipoproteins; IDL= intermediate density lipoproteins; L= large; LDL= low density liporpoteins; M= medium; S= small; VLDL= very low density lipoproteins; XL= very large; XS= very small; XXL= extremely large

Figure S3. Associations of metabolic biomarkers with risk of incident type 2 diabetes among 50,519 participants in the association analyses population

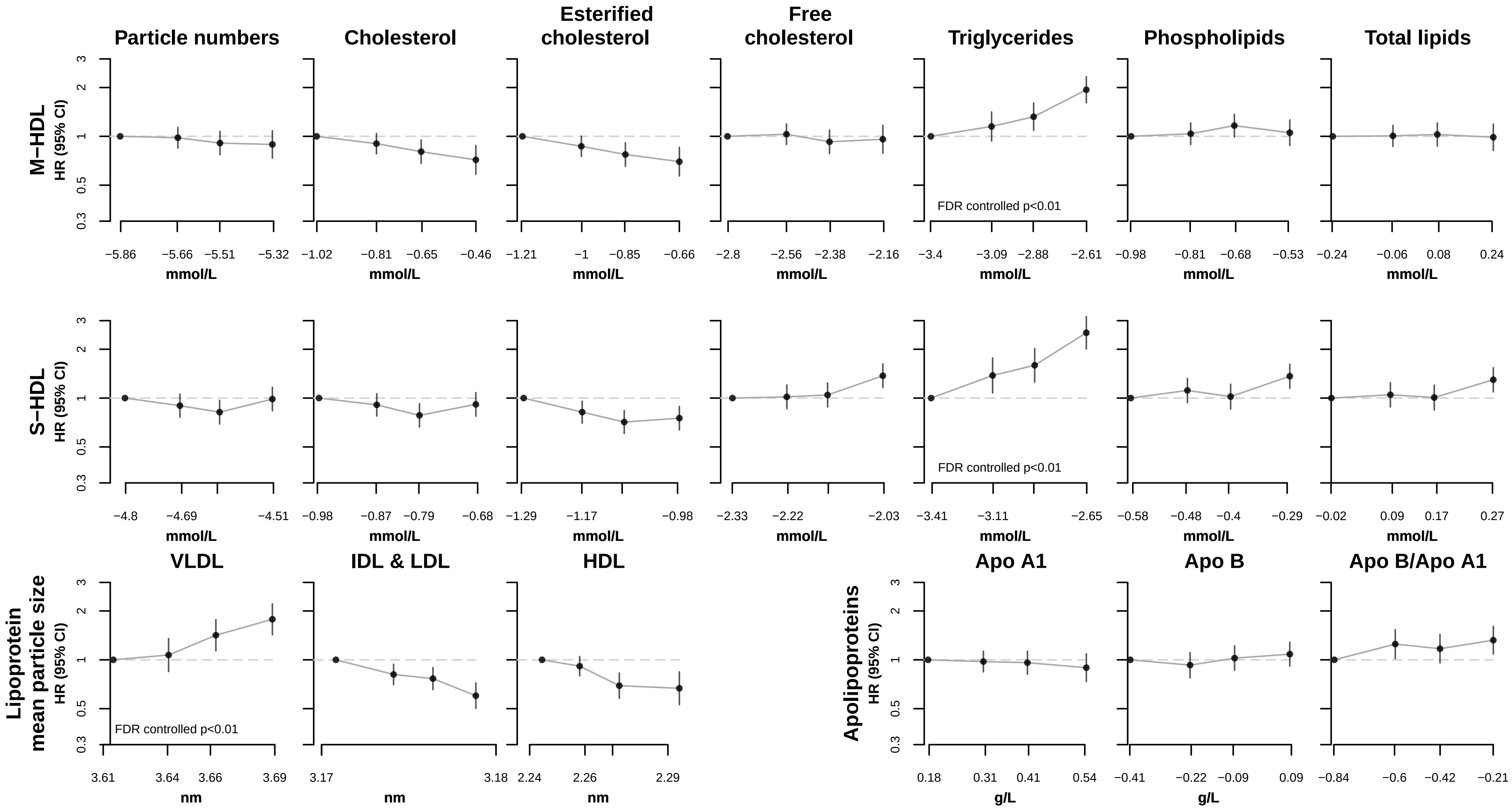

Hazard ratios (HR) stratified by age−at−risk and sex and adjusted for assessment centre, Townsend deprivation index, ethnicity, parental history of diabetes, smoking ,alcohol drinking, intake of whole grains, refined grains, fruit, vegetables, cheese, unprocessed red meat, processed meat, non−oily fish, oily fish, type of spread, coffee (regular and decaffeinated), tea, and dietary supplements, physical activity, body mass index, waist−to−hip ratio, fasting duration, and spectorometer.
Numbers on the x−axis correspond to median values within each quartile for each metabolic biomarker on the natural log scale.
Circles represent the HR and vertical lines indicate the 95% CI.
FDR controlled p values represent the p for trend across quartiles.
CM= chylomicrons; FA= fatty acids; HDL= high density lipoproteins; IDL= intermediate density lipoproteins; L= large; LDL= low density liporpoteins; M= medium; S= small; VLDL= very low density lipoproteins; XL= very large; XS= very small; XXL= extremely large

Figure S3. Associations of metabolic biomarkers with risk of incident type 2 diabetes among 50,519 participants in the association analyses population

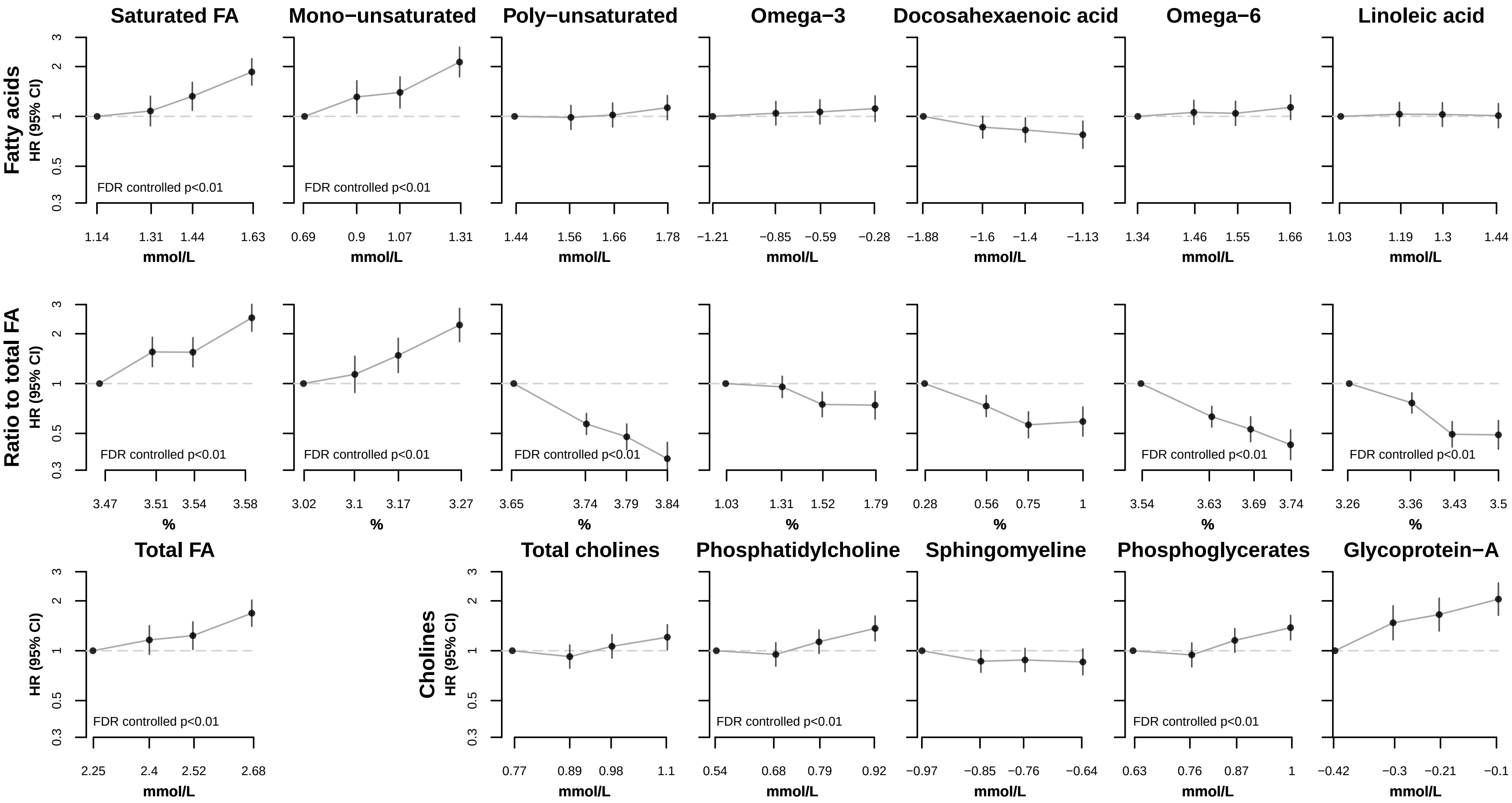

Hazard ratios (HR) stratified by age−at−risk and sex and adjusted for assessment centre, Townsend deprivation index, ethnicity, parental history of diabetes, smoking ,alcohol drinking, intake of whole grains, refined grains, fruit, vegetables, cheese, unprocessed red meat, processed meat, non−oily fish, oily fish, type of spread, coffee (regular and decaffeinated), tea, and dietary supplements, physical activity, body mass index, waist−to−hip ratio, fasting duration, and spectorometer.
Numbers on the x−axis correspond to median values within each quartile for each metabolic biomarker on the natural log scale.
Circles represent the HR and vertical lines indicate the 95% CI.
FDR controlled p values represent the p for trend across quartiles.
CM= chylomicrons; FA= fatty acids; HDL= high density lipoproteins; IDL= intermediate density lipoproteins; L= large; LDL= low density liporpoteins; M= medium; S= small; VLDL= very low density lipoproteins; XL= very large; XS= very small; XXL= extremely large

Figure S3. Associations of metabolic biomarkers with risk of incident type 2 diabetes among 50,519 participants in the association analyses population

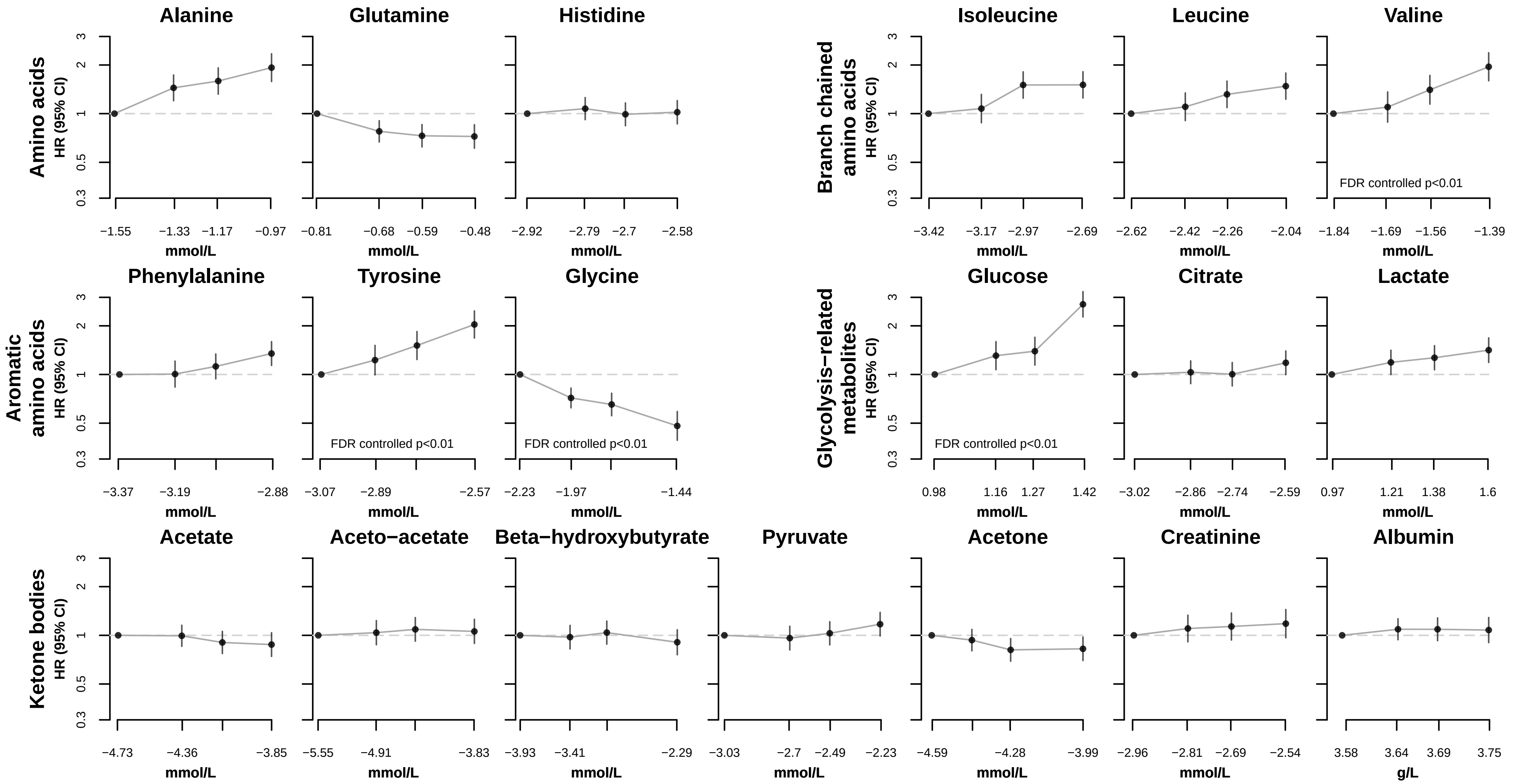

Hazard ratios (HR) stratified by age−at−risk and sex and adjusted for assessment centre, Townsend deprivation index, ethnicity, parental history of diabetes, smoking ,alcohol drinking, intake of whole grains, refined grains, fruit, vegetables, cheese, unprocessed red meat, processed meat, non−oily fish, oily fish, type of spread, coffee (regular and decaffeinated), tea, and dietary supplements, physical activity, body mass index, waist−to−hip ratio, fasting duration, and spectorometer.
Numbers on the x−axis correspond to median values within each quartile for each metabolic biomarker on the natural log scale.
Circles represent the HR and vertical lines indicate the 95% CI.
FDR controlled p values represent the p for trend across quartiles.
CM= chylomicrons; FA= fatty acids; HDL= high density lipoproteins; IDL= intermediate density lipoproteins; L= large; LDL= low density liporpoteins; M= medium; S= small; VLDL= very low density lipoproteins; XL= very large; XS= very small; XXL= extremely large

Figure S4. Associations of metabolic biomarkers with risk of incident type 2 diabetes by sex among 50,519 participants in the association analyses population

a) HR of incident T2D per 1−SD higher metabolic biomarker on the natural log scale

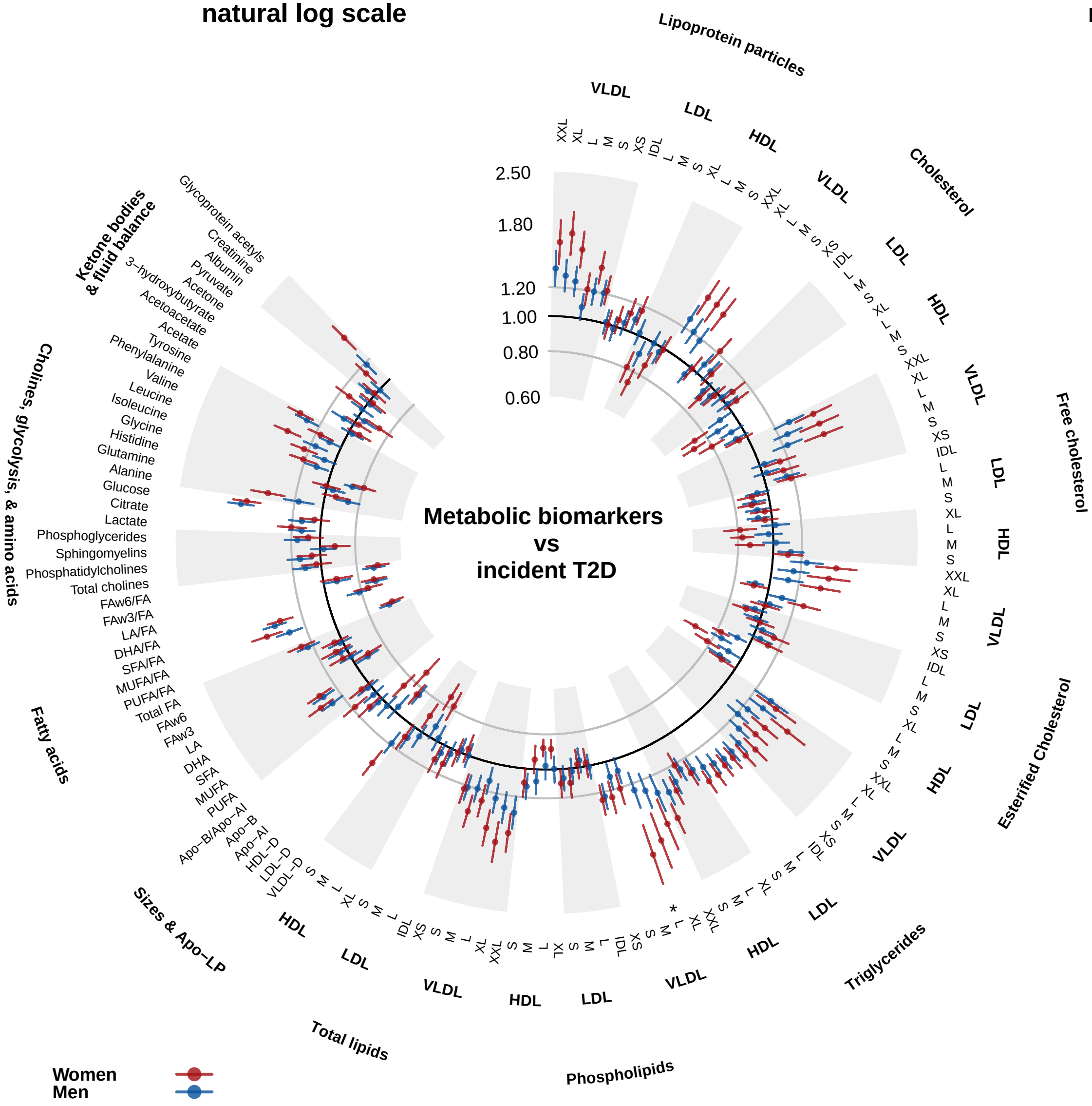

b) Effect size estimates for incident T2D per 1−SD higher metabolic biomarker on the natural log scale

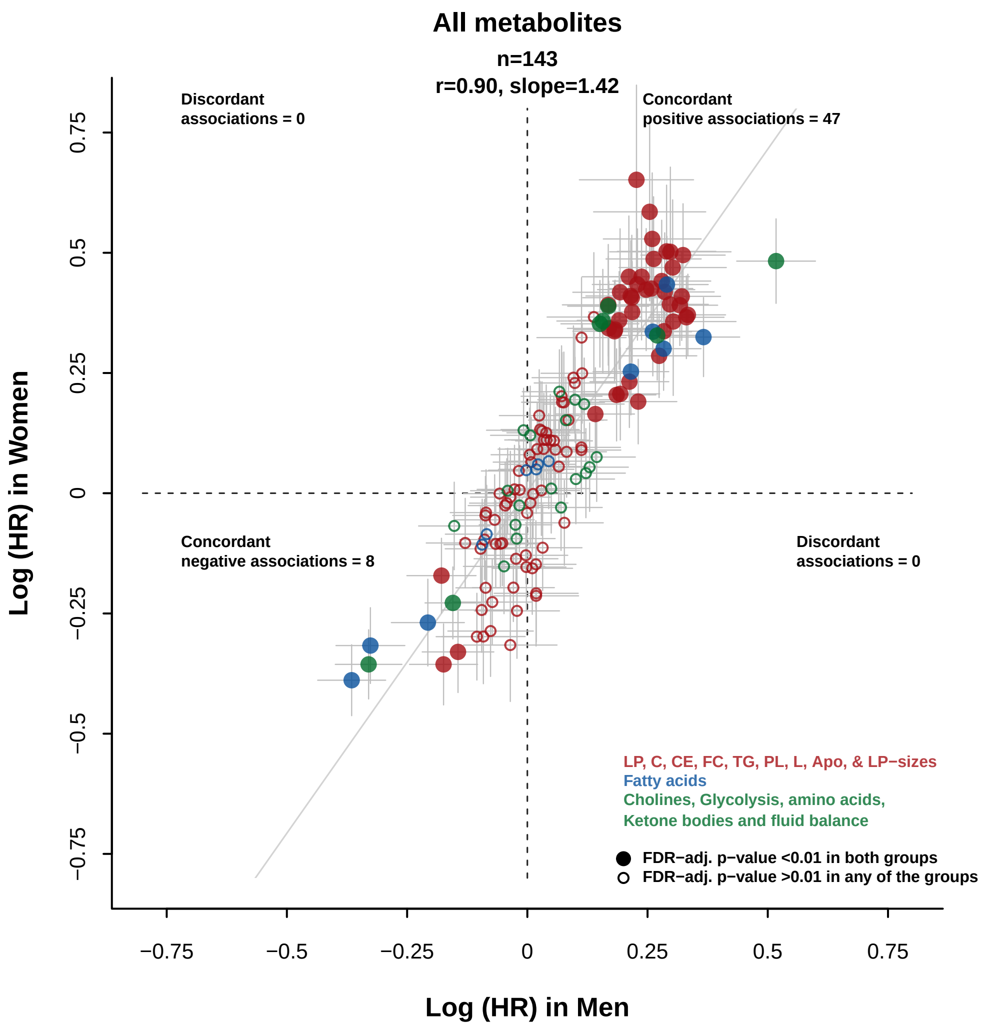

Hazard ratios (HR) stratified by age−at−risk and adjusted for assessment centre, Townsend deprivation index, ethnicity, parental history of diabetes, smoking, alcohol drinking, intake of whole grains, refined grains, fruit, vegetables, cheese, unprocessed red meat, processed meat, non−oily fish, oily fish, type of spread, coffee (regular and decaffeinated), tea, and dietary supplements, physical activity, body mass index, waist−to−hip ratio, fasting duration and spectrometer

Error bars represent 95% CIs

* p for heterogeneity <0.01 after controlling for false discovery rate

675 men and 536 women developed incident T2D

Apo=apolipoproteins; Apo−A1=apolipoprotein A1; Apo−B=apolipoprotein B; C=cholesterol; CE=esterified cholesterol; DHA=docosahexaenoic acid; FA=fatty acids; FAw3=omega−3 fatty acids; FAw6=omega−6 fatty acids; FC=free cholesterol; FDR=false discovery rate; HDL=high density lipoproteins; HDL−D=high density lipoprotein particle diameter; IDL=intermediate density lipoproteins; L=large; LA=linoleic acid; LDL=low density lipoproteins; LDL−D=low density lipoprotein particle diameter; LP=lipoprotein; M=medium; MUFA=monounsaturated fatty acids; PUFA=polyunsaturated fatty acids; S=small; SFA=saturated fatty acids; T2D=type 2 diabetes; TG=triglycerides; VLDL=very low density lipoproteins; VLDL−D=very low density lipoprotein particle diameter; XL=very large; XS=very small; XXL=extremely large

Figure S5. Associations of metabolic biomarkers with risk of incident type 2 diabetes by age among 50,519 participants in the association analyses population

a) HR of incident T2D per 1−SD higher metabolic biomarker on the natural log scale

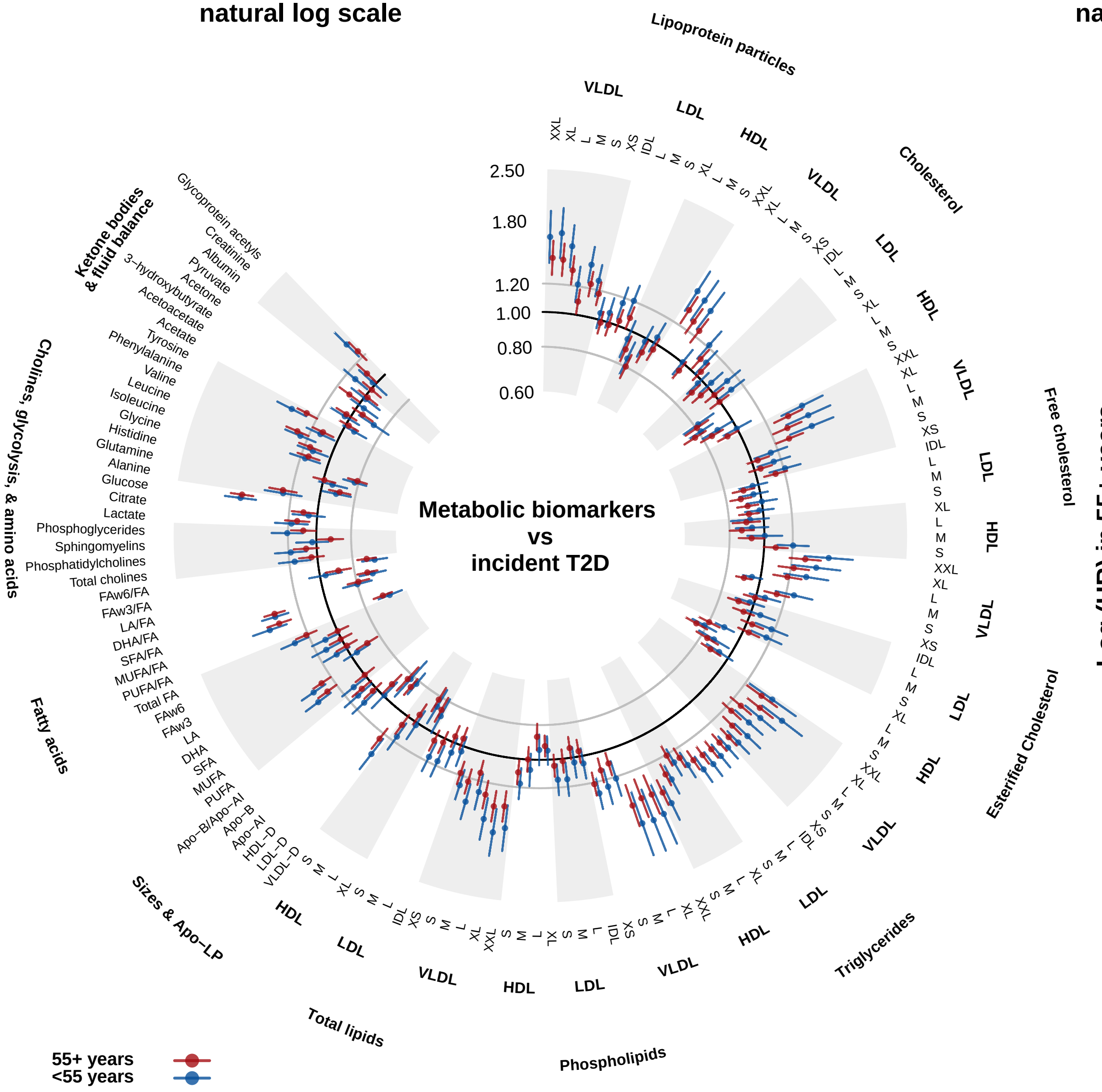

b) Effect size estimates for incident T2D per 1−SD higher metabolic biomarker on the natural log scale

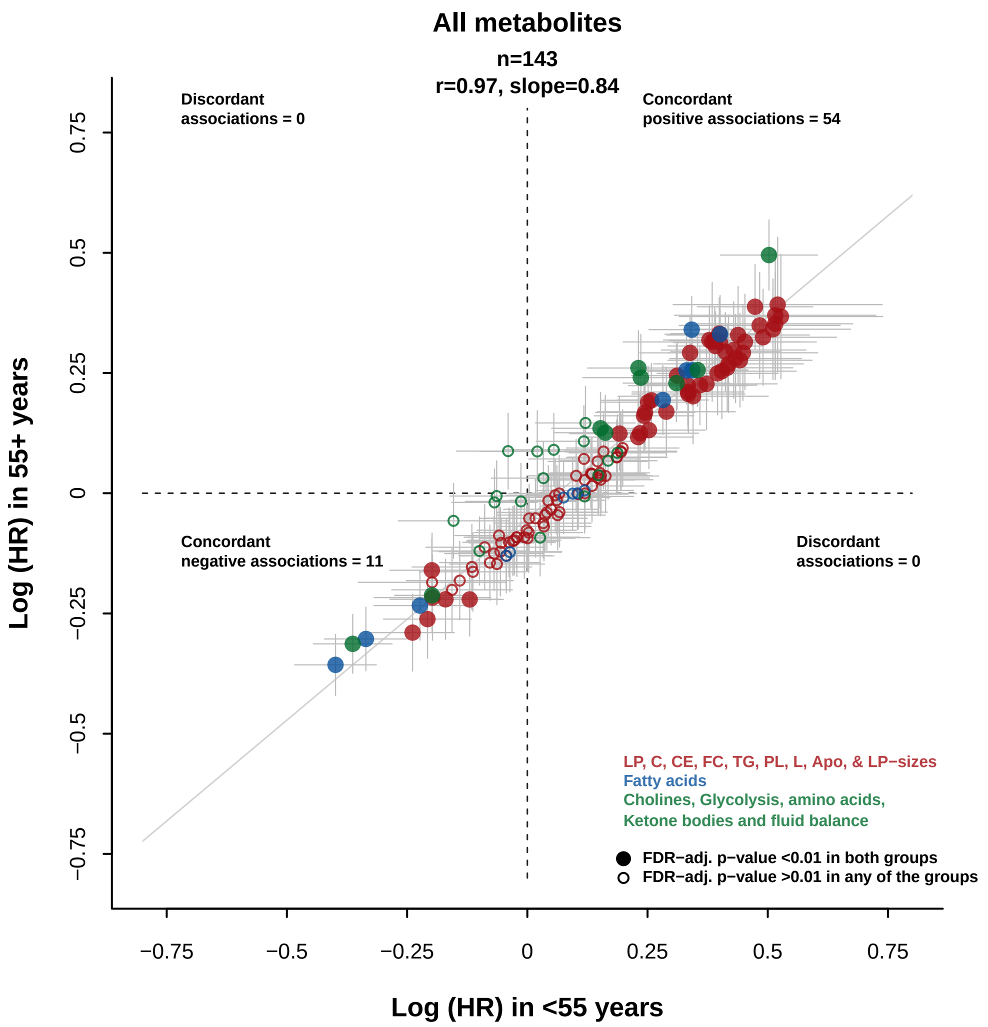

Hazard ratios (HR) stratified by age−at−risk and adjusted for assessment centre, Townsend deprivation index, ethnicity, parental history of diabetes, smoking, alcohol drinking, intake of whole grains, refined grains, fruit, vegetables, cheese, unprocessed red meat, processed meat, non−oily fish, oily fish, type of spread, coffee (regular and decaffeinated), tea, and dietary supplements, physical activity, body mass index, waist−to−hip ratio, fasting duration and spectrometer

Error bars represent 95% CIs

* p for heterogeneity <0.01 after controlling for false discovery rate

434 participants <55 years at baseline and 777 participants 55+ years at baseline developed incident T2D

Apo=apolipoproteins; Apo−A1=apolipoprotein A1; Apo−B=apolipoprotein B; C=cholesterol; CE=esterified cholesterol; DHA=docosahexaenoic acid; FA=fatty acids; FAw3=omega−3 fatty acids; FAw6=omega−6 fatty acids; FC=free cholesterol; FDR=false discovery rate; HDL=high density lipoproteins; HDL−D=high density lipoprotein particle diameter; IDL=intermediate density lipoproteins; L=large; LA=linoleic acid; LDL=low density lipoproteins; LDL−D=low density lipoprotein particle diameter; LP=lipoprotein; M=medium; MUFA=monounsaturated fatty acids; PUFA=polyunsaturated fatty acids; S=small; SFA=saturated fatty acids; T2D=type 2 diabetes; TG=triglycerides; VLDL=very low density lipoproteins; VLDL−D=very low density lipoprotein particle diameter; XL=very large; XS=very small; XXL=extremely large

Figure S6. Associations of metabolic biomarkers with risk of incident type 2 diabetes excluding the first three years of follow−up in the association analyses population

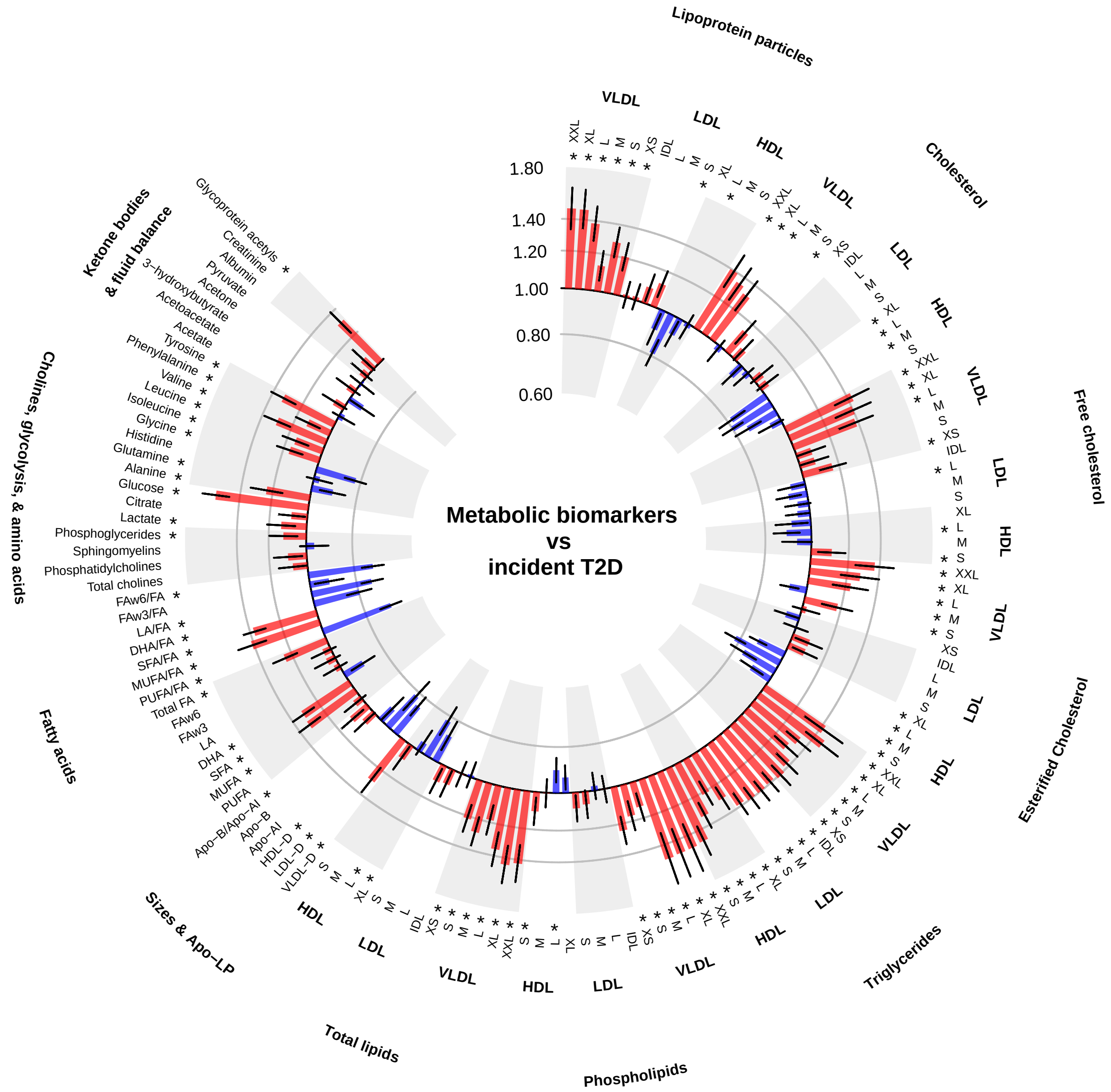

Hazard ratios are presented per 1−SD higher metabolic biomarkers on the natural log scale, stratified by age−at−risk and sex and adjusted for assessment centre, Townsend deprivation index, ethnicity, parental history of diabetes, smoking, alcohol drinking, intake of whole grains, refined grains, fruit, vegetables, cheese, unprocessed red meat, processed meat, non−oily fish, oily fish, type of spread, coffee (regular and decaffeinated), tea, and dietary supplements, physical activity, body mass index, waist−to−hip ratio, fasting duration, and spectrometer

Error bars represent 95% CIs

* false discovery rate controlled p<0.01

Apo−A1=apolipoprotein A1; Apo−B=apolipoprotein B; DHA=docosahexaenoic acid; FA=fatty acids; FAw3=omega−3 fatty acids; FAw6=omega−6 fatty acids; HDL=high density lipoproteins; HDL−D=high density lipoprotein particle diameter; IDL=intermediate density lipoproteins; L=large; LA=linoleic acid; LDL=low density lipoproteins; LDL−D=low density lipoprotein particle diameter; LP=lipoprotein; M=medium; MUFA=monounsaturated fatty acids; PUFA=polyunsaturated fatty acids; S=small; SFA=saturated fatty acids; T2D=type 2 diabetes; VLDL=very low density lipoproteins; VLDL−D=very low density lipoprotein particle diameter; XL=very large; XS=very small; XXL=extremely large

Figure S7. Importance of the first 20 metabolic biomarker principal components

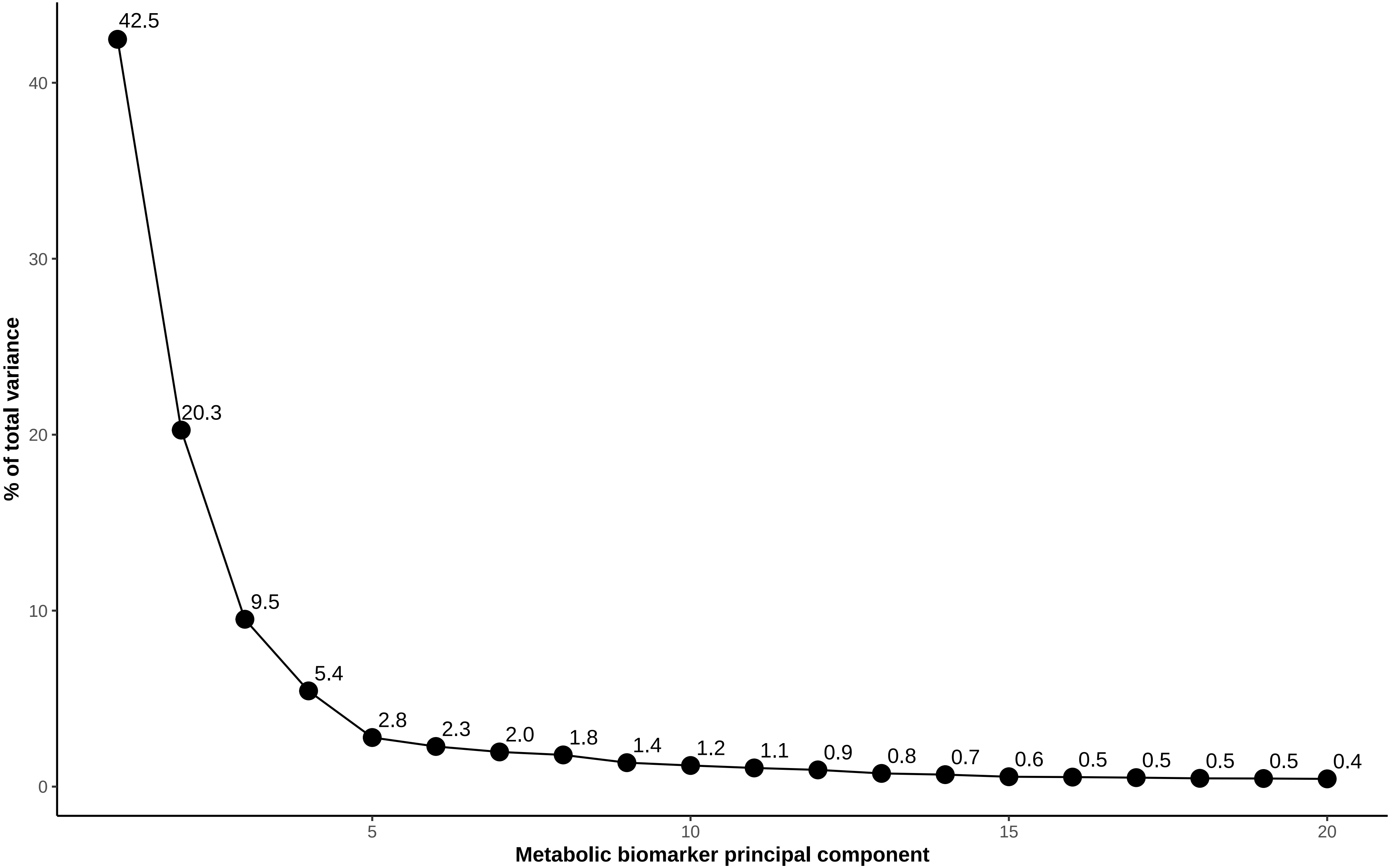

Numbers adjacent to the points are the percentages of total variance in individual biomarkers explained by each principal component.

Figure S8. Characterisation of the first 11 metabolic biomarker principal components among 65,684 participants in the risk prediction population

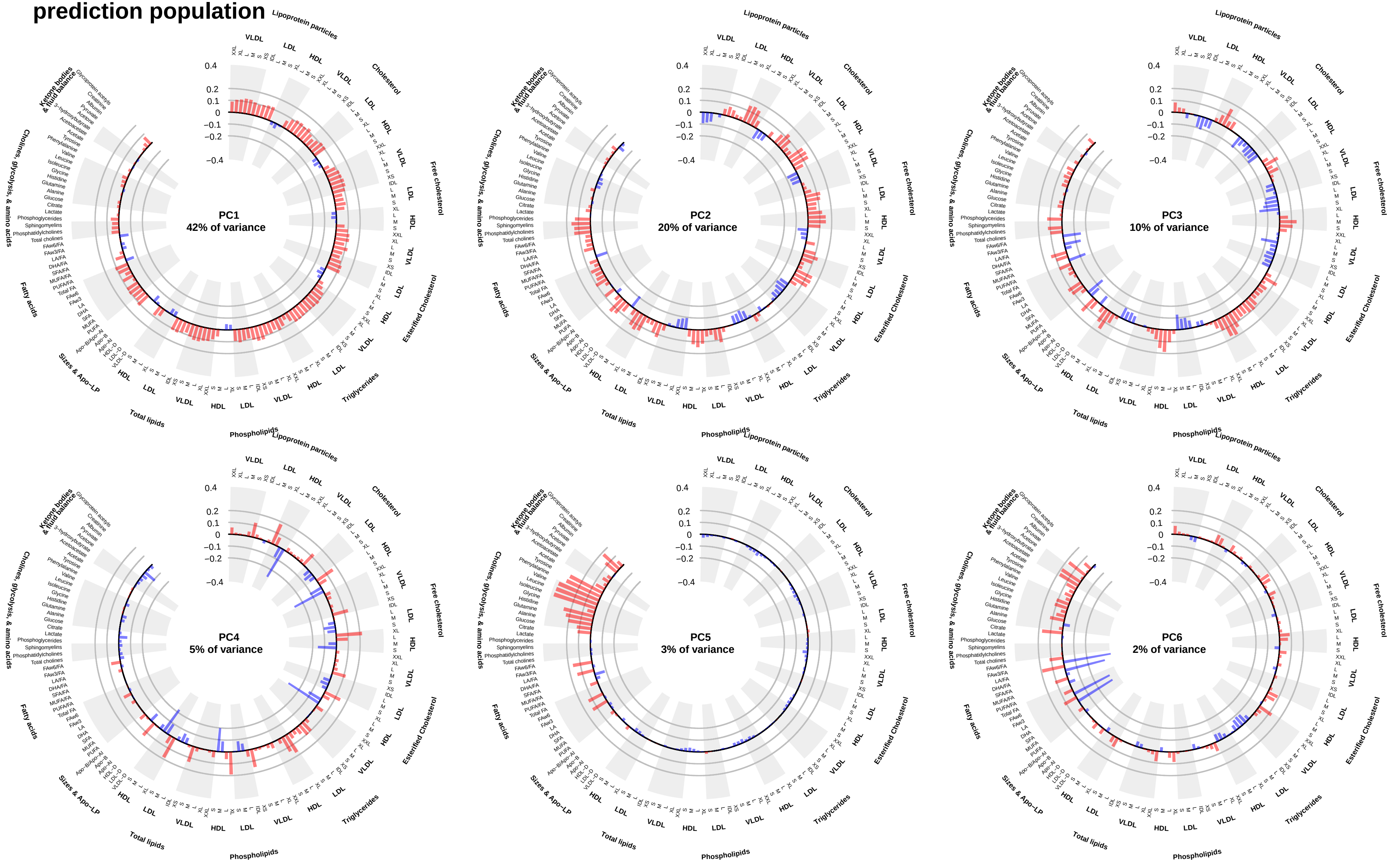

Factor loadings for each principal component are presented, representing the coefficients of the metabolic biomarkers from which each principal component was calculated. The first 6 principal components explain 83 % of the variance. Apo−A1=apolipoprotein A1; Apo−B=apolipoprotein B;DHA=docosahexaenoic acid; FA=fatty acids; FAw3=omega−3 fatty acids; FAw6=omega−6 fatty acids; HDL=high density lipoproteins; HDL−D=high density lipoprotein particle diameter; IDL=intermediate density lipoproteins; L=large; LA=linoleic acid; LDL=low density lipoproteins; LDL−D=low density lipoprotein particle diameter; LP=lipoprotein; M=medium; MUFA=monounsaturated fatty acids; PC1=principal component 1; PC2=principal component 2; PC3=principal component 3; PC4=principal component 4; PC5=principal component 5; PC6=principal component 6; PC7=principal component 7; PC8=principal component 8; PC9=principal component 9; PC10=principal component 10; PC11=principal component 11; PUFA=polyunsaturated fatty acids; S=small; SFA=saturated fatty acids; T2D=type 2 diabetes; VLDL=very low density lipoproteins; VLDL−D=very low density lipoprotein particle diameter; XL=very large; XS=very small; XXL=extremely large

Figure S8. Characterisation of the first 11 metabolic biomarker principal components among 65,684 participants in the risk prediction population

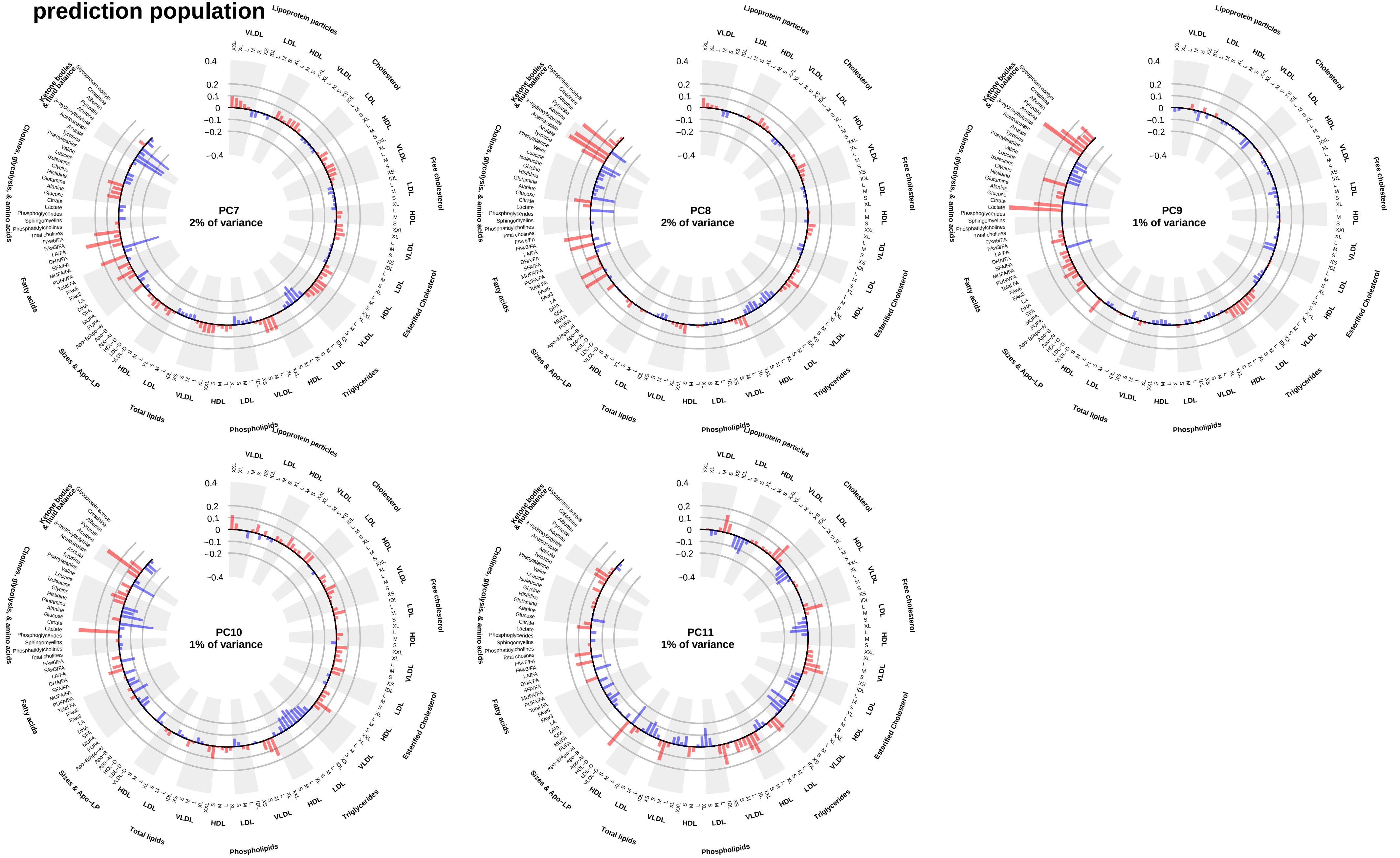

Factor loadings for each principal component are presented, representing the coefficients of the metabolic biomarkers from which each principal component was calculated. The first 11 principal components explain 90 % of the variance. Apo−A1=apolipoprotein A1; Apo−B=apolipoprotein B; DHA=docosahexaenoic acid; FA=fatty acids; FAw3=omega−3 fatty acids; FAw6=omega−6 fatty acids; HDL=high density lipoproteins; HDL−D=high density lipoprotein particle diameter; IDL=intermediate density lipoproteins; L=large; LA=linoleic acid; LDL=low density lipoproteins; LDL−D=low density lipoprotein particle diameter; LP=lipoprotein; M=medium; MUFA=monounsaturated fatty acids; PC1=principal component 1; PC2=principal component 2; PC3=principal component 3; PC4=principal component 4; PC5=principal component 5; PC6=principal component 6; PC7=principal component 7; PC8=principal component 8; PC9=principal component 9; PC10=principal component 10; PC11=principal component 11; PUFA=polyunsaturated fatty acids; S=small; SFA=saturated fatty acids; T2D=type 2 diabetes; VLDL=very low density lipoproteins; VLDL−D=very low density lipoprotein particle diameter; XL=very large; XS=very small; XXL=extremely large

Figure S9. Comparison of biomarkers measured by NMR and routine clinical chemistry assays among 65,684 participants in the risk prediction population

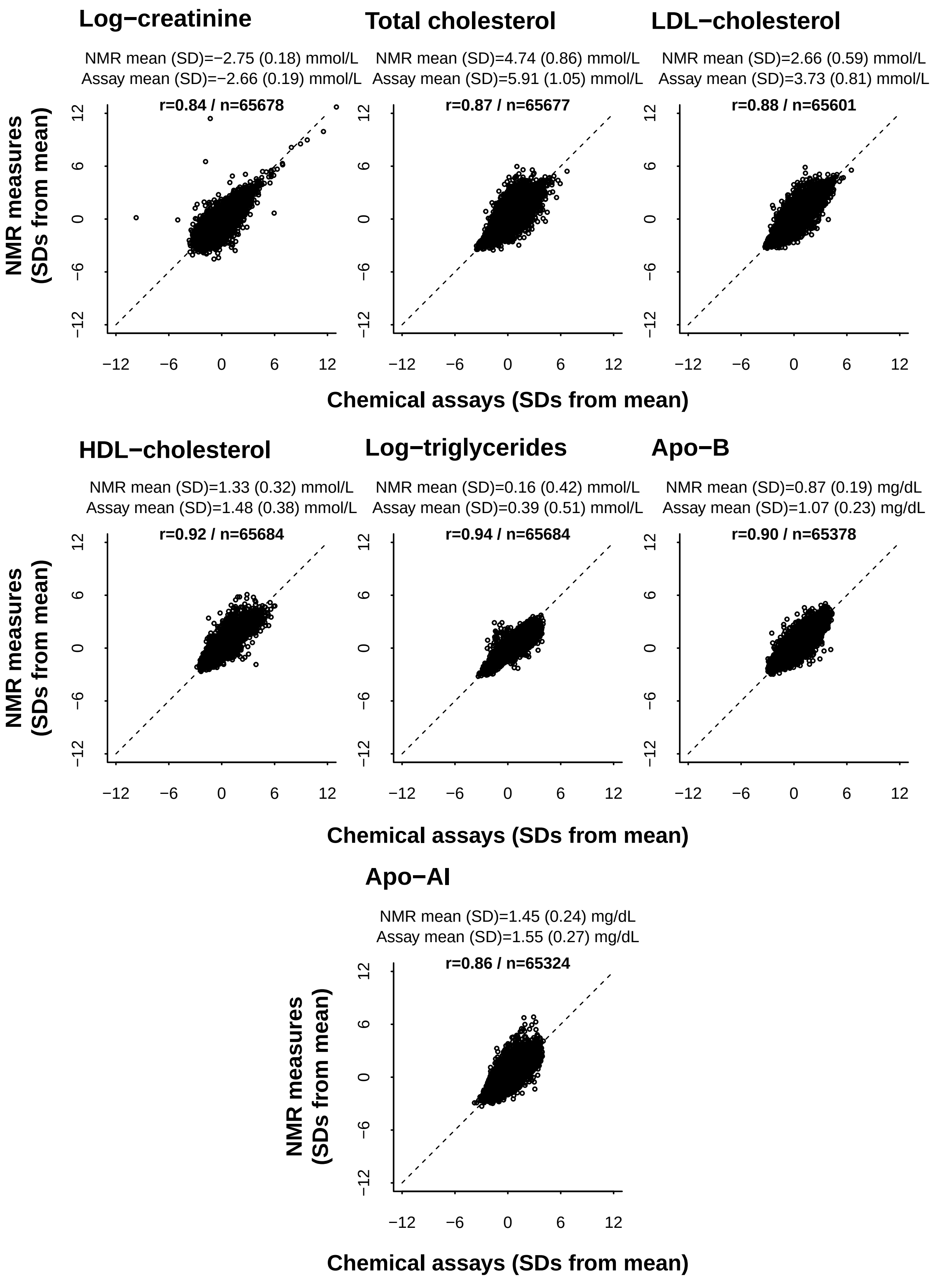

r = Pearson's correlation coefficient between NMR measures and chemical assays.
Clinical chemistry assays were conducted on serum samples. NMR profiling was conducted on EDTA plasma samples from different aliquots.
For the cholesterol chemical assay, LDL−cholesterol was directly measured using a technique that first solubilises and extracts CM−, VLDL− and HDL−carried cholesterol, and then quantifies the remaining cholesterol carried by both IDL and LDL particles. For NMR measures, LDL−cholesterol corresponds to the sum of all the NMR−derived measures of both esterified and free cholesterol carried by both IDL and LDL particles.
Apo= apolipoprotein; NMR= nuclear magnetic resonance
